# Postoperative elective pelvic nodal irradiation compared to prostate bed irradiation in locally advanced prostate cancer – a retrospective analysis of dose-escalated patients

**DOI:** 10.1186/s13014-019-1301-5

**Published:** 2019-06-07

**Authors:** Carola Link, Patrick Honeck, Akiko Makabe, Frank Anton Giordano, Christian Bolenz, Joerg Schaefer, Markus Bohrer, Frank Lohr, Frederik Wenz, Daniel Buergy

**Affiliations:** 1 Department of Radiation Oncology, Universitätsmedizin Mannheim, Medical Faculty Mannheim, Heidelberg University, Mannheim, Germany; 2 Department of Urology, University Medical Center Mannheim, Medical Faculty Mannheim, University of Heidelberg, Mannheim, Germany; 3 Department of Urology, University of Ulm, Ulm, Germany; 4 Struttura Complessa di Radioterapia, Dipartimento di Oncologia, Azienda Universitario-Ospedaliera, Policlinico, Modena, Italy; 5 Freiburg Medical Center, Freiburg, Germany; 6 Heinrich-Lanz-Center for Digital Medicine, Medical Faculty Mannheim, University of Heidelberg, Mannheim, Germany

**Keywords:** Prostate carcinoma, Elective nodal irradiation, Salvage radiotherapy, Adjuvant radiotherapy

## Abstract

**Background:**

It is uncertain if whole-pelvic irradiation (WPRT) in addition to dose-escalated prostate bed irradiation (PBRT) improves biochemical progression-free survival (bPFS) after prostatectomy for locally advanced tumors. This study was initiated to analyze if WPRT is associated with bPFS in a patient cohort with dose-escalated (> 70 Gy) PBRT.

**Methods:**

Patients with locally advanced, node-negative prostate carcinoma who had PBRT with or without WPRT after prostatectomy between 2009 and 2017 were retrospectively analyzed. A simultaneous integrated boost with equivalent-doses-in-2-Gy-fractions (EQD-2) of 79.29 Gy or 71.43 Gy to the prostate bed was applied in patients with margin-positive (or detectable) and margin-negative/undetectable tumors, respectively. WPRT (44 Gy) was offered to patients at an increased risk of lymph node metastases.

**Results:**

Forty-three patients with PBRT/WPRT and 77 with PBRT-only were identified. Baseline imbalances included shorter surgery-radiotherapy intervals (S-RT-Intervals) and fewer resected lymph nodes in the WPRT group. WPRT was significantly associated with better bPFS in univariate (*p* = 0.032) and multivariate models (HR = 0.484, *p* = 0.015). Subgroup analysis indicated a benefit of WPRT (*p* = 0.029) in patients treated with rising PSA values who mostly had negative margins (74.1%); WPRT was not associated with a longer bPFS in the postoperative setting with almost exclusively positive margins (96.8%).

**Conclusion:**

We observed a longer bPFS after WPRT compared to PBRT in patients with locally advanced prostate carcinoma who underwent dose-escalated radiotherapy. In subset analyses, the association was only observed in patients with rising PSA values but not in patients with non-salvage postoperative radiotherapy for positive margins.

**Electronic supplementary material:**

The online version of this article (10.1186/s13014-019-1301-5) contains supplementary material, which is available to authorized users.

## Background

Post-prostatectomy radiotherapy can be given as adjuvant [1–3] or salvage [4] treatment with or without androgen-deprivation therapy (ADT) [5, 6]. The addition of ADT has just recently been shown to improve biochemical progression-free survival (bPFS) [5] and also overall survival (OS) after longer follow-up [6] when added to moderate-dose radiation therapy (66 and 64.8 Gy, respectively, at the lower end of most recent EU and US guideline recommendations (≥66 and ≥ 64–65 Gy [7, 8])). Although an optimal dose has not been established for salvage or adjuvant radiotherapy, some series indicate that doses > 70 Gy might improve bPFS [7–12]; however, dose escalation was associated with increased risk of toxicity even in recent series using highly conformal techniques such as intensity-modulated radiotherapy (IMRT) [13–15].

Aside from dose escalation and addition of ADT, there is ongoing discussion about the effect of elective whole-pelvic radiotherapy (WPRT). Despite negative studies in the primary setting [16, 17] and an absence of evidence in the post-prostatectomy situation at the time when the survey was conducted, WPRT has been considered by 74% of radiation oncologists in the salvage setting [18]. Interim results from the 3-arm randomized NRG-Oncology/RTOG-0534 trial support this approach; the study recently reported a bPFS benefit in the salvage setting by adding ADT or ADT plus WPRT to prostate bed (“fossa-only”; PBRT) irradiation [19].

We primarily conducted this study to analyze outcomes of patients with locally advanced but lymph-node-negative tumors (T3–4/N0) treated with two standardized, dose-escalated (71.43–79.29 Gy equivalent-doses-in-2-Gy-fractions, EQD-2) post-prostatectomy radiotherapy strategies with or without WPRT (44 Gy, EQD-2 with volumes in line with RTOG recommendations [20]). Supplementary analyses were done as a pooled cohort with all node-negative patients (including T1–2/N0) and with node-positive patients (N1); however, both groups were not included in the main model due to extreme imbalances of PBRT and WPRT usage.

## Methods

### Patients

Following institutional review board approval (2018-509 N-MA), we analyzed charts of 244 patients that were treated between 01/2009 and 07/2017 (≥6 months follow-up) with postoperative (salvage or adjuvant) radiotherapy to the prostate bed region (for in- and exclusion criteria see Additional file 1: Figure S1). In short, patients with locally advanced, node-negative (T3–4/N0) cancers were eligible in the post-prostatectomy setting irrespective of laparoscopic/robotic or open surgery or extent of lymph node resection. Patients with localized tumors (T1–2/N0) were not included in the main model as only 8 out of 52 patients had received WPRT (15.4%); whereas almost all patients with node-positive disease had received WPRT (38 out of 40; 95%); therefore, pooled analyses including those two groups were deemed exploratory and are reported in the supplementary appendix.

### Radiotherapy

The clinical target volume-1 (CTV-1) included prostate bed, bladder neck, and urethral anastomosis regions in accordance with the Princess Margaret Hospital guidelines [21]. The seminal vesicle bed was always included in CTV-1 [22]. CTV-2 included prostate bed, bladder neck and anastomosis but not necessarily the seminal vesicle bed; both CTVs included localizable disease. Out of CTV-1/2, PTV-1/2 were created using a margin of 3–8 mm. PTV-1 and PTV-2 were always irradiated with 60 Gy in 30 fractions. Patients without localizable tumors then received an additional dose of 6–7 Gy to PTV-1 plus 10 Gy as a 4-fraction simultaneous integrated boost (SIB) to PTV-2. Patients with detectable tumor manifestations (positive imaging or positive margins) received a 5-fraction SIB of 11–12 Gy to PTV-1 and 15 Gy to PTV-2. Assuming an α/β of 1.5, this would result in an EQD-2 to PTV-1 of 73.37 or 66.5 Gy and PTV-2 doses of 79.29 Gy (“high-dose cohort”) or 71.43 Gy (“lower-dose cohort”) for patients with detectable and undetectable disease, respectively.

SIB fractions were always applied as IMRT (or Volumetric Modulated Arc Therapy, VMAT) using daily image-guidance (IGRT) with cone-beam computed tomography (CBCT). The initial 60 Gy were either given as a 3D-conformal or as an IMRT/VMAT-based approach.

Elective nodal irradiation with 44 Gy in 22 fractions was offered to patients with increased risk of nodal involvement. The latter was determined using surgically removed nodes: In case of N0 with ≥10 negative nodes, pelvic radiation was not recommended. In case of < 10 evaluable nodes and a Roach score [23] of ≥15 (before 2012) or ≥ 25 after adjustment in 2012, elective pelvic radiotherapy was offered to patients. For elderly patients (≥70 years) WPRT was offered in case geriatric assessment yielded a favorable profile; otherwise, PBRT was recommended using high- or lower-dose approaches as described. Due to the lack of definitive evidence, we always accepted the patient’s decision in favor or against pelvic radiotherapy after careful discussion of the evidence.

Pelvic target volumes were defined similar to the RTOG recommendations [20]; briefly, the superior border was the L5/S1 interspace (in case of positive nodes: L4/L5), the pubic symphysis was the anterior, and the lower aspect of the obturator foramen the inferior border. Dose constraints were in line with QUANTEC [24] recommendations for bladder, rectal, and bowel doses.

Concurrent ADT was used on an individual basis in our department prior to the publication of the GETUG-AFU-16 [5] trial and the RTOG-9601 [6] study. After publication of the results, we discussed the possibility of concurrent ADT with all patients who had postoperative radiotherapy; for this analysis, patients who had received concurrent ADT were not excluded.

### Statistical calculations

All analyses were performed using SPSS (V15.0), or “R”, a language and environment for statistical computing. Survival curves were computed using the Kaplan-Meier approach; log-rank tests were used to calculate univariate associations with survival. Univariately associated variables entered a multivariate stepwise Cox regression model to identify independent predictors.

To address (unrelated) death as a confounder, bPFS analyses were complemented by freedom from biochemical failure (FFBF) analyses in which death events were censored. This was done to account for the increased risk of death in elderly patients as a potential confounder for bPFS.

## Results

### Patient characteristics

Baseline characteristics and differences between WPRT (*n* = 43), and PBRT (*n* = 77) patients are shown in Table 1. The median follow-up time was 62.2 months (range: 8.5–106.5 months). In the whole cohort of 120 locally advanced patients, average bPFS was 54.6 (95%-CI 46.3–62.9; median: 46.9) months and FFBF was 56.2 (47.8–64.6; median: 51.4) months.

**Table 1:** Baseline characteristics of 120 patients with locally advanced tumors who received fossa-only (*n* = 77) or elective pelvic nodal irradiation (WPRT; *n* = 43)

Baseline Characteristics	Fossa-only (*n* = 77)	WPRT (*n* = 43)	Difference
Age at radiation therapy in years			
Mean	66.9	66.9	*p* = 0.98
Standard deviation	7.5	6.5	
Initial PSA value in ng/ml			
Mean	18.1	18.6	*p* = 0.927
Standard deviation	26.8	18.0	
Gleason score (surgical specimens, No., percentage)			
Gleason 6	2 (2.6%)	0	
Gleason 7a	15 (19.5%)	9 (20.9%)	
Gleason 7b	30 (39.0%)	10 (23.3%)	*p* = 0.087^§^
Gleason 8	7 (9.1%)	4 (9.3%)	
Gleason 9	22 (28.6%)	19 (44.2%)	
Gleason 10	1 (1.3%)	1 (2.3%)	
Surgical margin (No., percentage)			
R0	29 (37.7%)	16 (37.2%)	*p* = 1.0
R1	48 (62.3%)	27 (62.8%)	
R2	–	–	
Dose level (Gy, prescribed dose, EQD-2 to PTV-2)			
Lower-dose (71.43 Gy)	29 (37.7%)	13 (30.2%)	*p* = 0.433
High-dose (79.29 Gy)	48 (62.3%)	30 (69.8%)	
Time from surgery to radiation therapy (S-RT-Interval in months)			
Mean	13.3	6.1	p = 0.003^#^
Median	5.1	3.6	
Standard deviation	18.7	6.2	
Salvage or postoperative (adjuvant or R1) treatment indication (No., percentage)			
Salvage	41 (53.2%)	17 (39.5%)	*p* = 0.184
Postoperative	36 (46.8%)	26 (60.5%)	
Roach scores			
Mean	28.9	32.8	*p* = 0.27
Standard deviation	20.8	15.0	
Concurrent androgen deprivation therapy (ADT)			
Yes	7 (9.1%)	6 (14%)	*p* = 0.541
No	70 (90.9%)	37 (86%)	
Pathologically identified number of lymph nodes (all N0)			
Mean	11.0	5.9	*p* < 0.001^†^
Standard deviation	6.5	3.9	
≥ 10 nodes identified	42 (54.5%)	2 (4.7%)	
< 10 nodes identified	35 (45.5%)	41 (95.3%)	
Post-surgery PSA at start of radiotherapy (if available) in ng/ml			
Mean	0.73	0.71	*p* = 0.958
Standard deviation	1.62	1.66	
Laparoscopic or retropubic surgery			
Laparoscopic	31 (40.3%)	23 (53.5%)	*p* = 0.184
Retropubic	46 (59.7%)	20 (46.5%)	

^§^Fisher’s exact test, 2-sided for Gleason scores dichotomized at 7 (6–7 vs. 8–10); one-sided testing results in *p* = 0.056 for an increased Gleason score in the WPRT group

^#^The baseline imbalance was significant using the independent samples T-test; imbalance was mainly due to outliers (non-parametric testing did not show a significant difference: *p* = 0.07 for Mann-Whitney U-Test

^†^WPRT was not routinely recommended in patients with ≥10 lymph nodes resected and only performed in two cases after careful discussion with patients. If no lymph node dissection was performed, the number was scored as zero

### Radiotherapy factors univariately associated with bPFS or FFBF

**Fig. 1):**
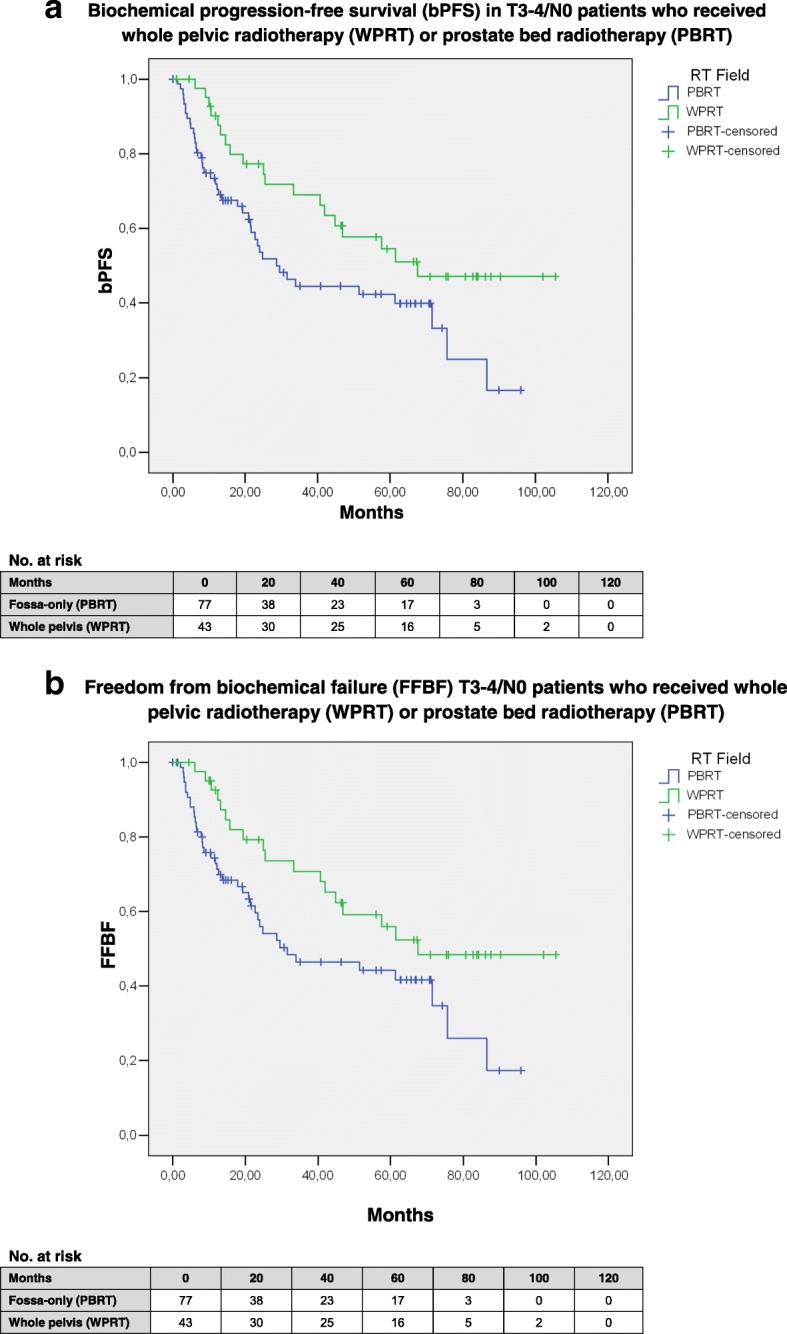
Biochemical progression-free survival (bPFS; 1**a**) and freedom from biochemical failure (FFBF; 1**b**) after whole-pelvic radiotherapy (WPRT) compared to fossa-only radiotherapy (PBRT) in patients with locally advanced node-negative prostate carcinomas after prostatectomy. Albeit not significantly different, the PBRT group included a higher percentage of patients with salvage indication (53.2%) compared to the WPRT group (39.5%); furthermore, patients were treated earlier after surgery in the WPRT group (6.1 months vs. 13.3 months, *p* = 0.003) likely confounding these results in favor of WPRT. Other baseline imbalances are shown in Table 1. **a** Mean bPFS in the WPRT group was 66.4 months (95%-CI: 53.6–79.2 months; median: 67.6 months) and significantly longer (*p* = 0.032) compared to 44.7 months (95%-CI: 35.4–54.0 months; median: 28.7 months) in the PBRT group. **b** Mean FFBF in the WPRT group was 67.9 months (95%-CI: 55.1–80.7 months; median: 67.6 months) and significantly increased (*p* = 0.033) compared to 46.1 months (95%-CI: 36.7–55.5 months; median: 31.5 months) in the PBRT group

WPRT was significantly associated with bPFS (*p* = 0.032) and FFBF (*p* = 0.033) in univariate analysis (see Fig. 1a-b and Table 1 for details on baseline imbalances).

**Fig. 2):**
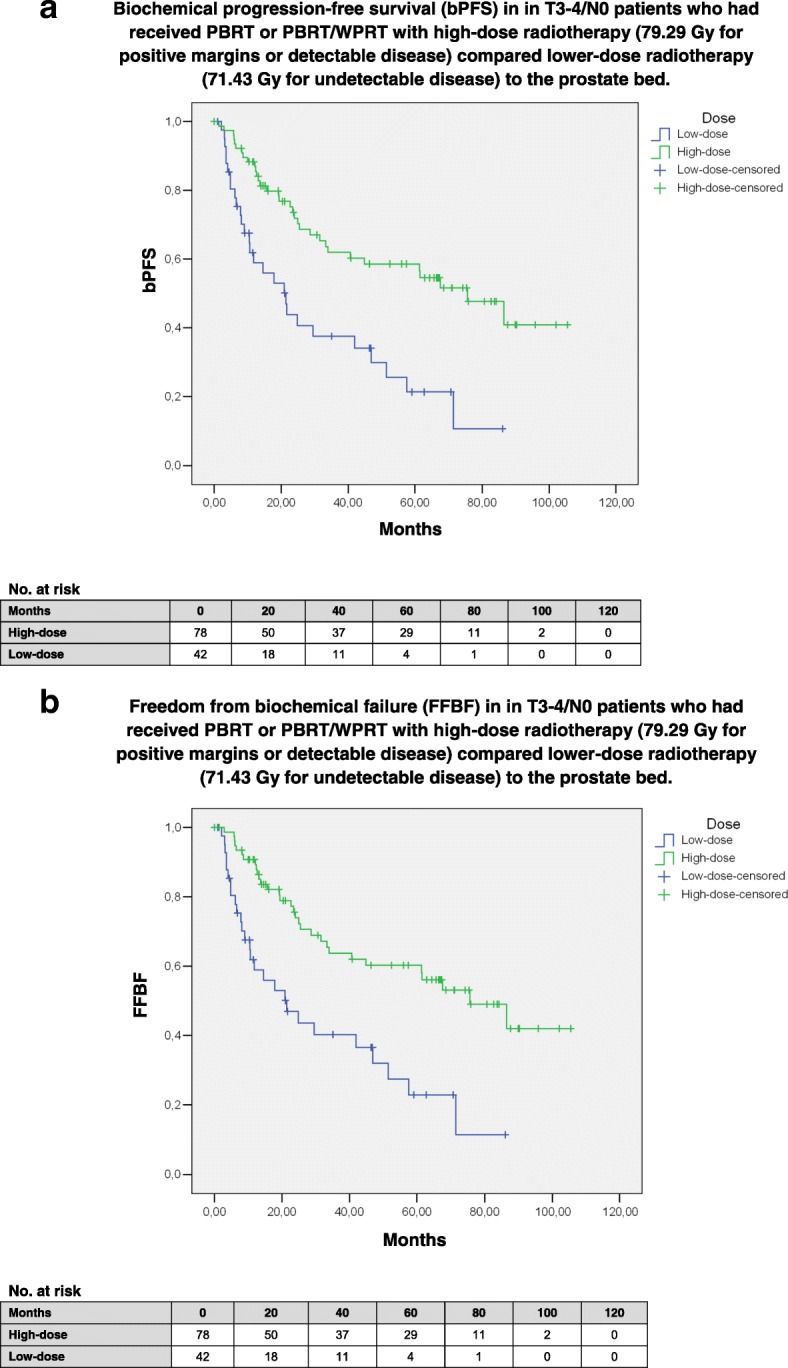
Biochemical progression-free survival (bPFS; 2**a**) and freedom from biochemical failure (FFBF; 2**b**) after high-dose radiotherapy (79.29 Gy) compared to lower-dose irradiation (71.43 Gy) in patients with locally advanced node-negative prostate carcinomas after prostatectomy who received PBRT-only or WPRT/PBRT. 93.6% of high-dose patients had positive margins and 97.3% of margin-positive patients had high-dose irradiation. Furthermore, 95.2% of patients with lower-dose radiotherapy had salvage treatment compared to 23.1% of patients with high-dose radiotherapy (*p* < 0.001). Therefore, the benefit in bPFS and FFBF cannot be assigned to either positive margins, higher doses or treatment indication as it is highly likely that all factors independently contributed to the better outcomes. A multivariate model could not be applied due to the high overlap of high-dose patients with positive margins. **a** Mean bPFS in the high-dose (mostly R1/postoperative) group was 64.4 months (95%-CI: 54.3–74.5 months; median: 75.7 months) and significantly longer (*p* < 0.001) compared to 32.2 months (95%-CI: 22.3–42.0 months; median: 21.4 months) in the low-dose (R0/salvage) group. **b** Mean FFBF in the high-dose (R1/postoperative) group was 66.1 months (95%-CI: 56.0–76.2 months; median: 75.7 months) and significantly longer (*p* < 0.001) compared to 33.4 months (95%-CI: 23.3–43.5 months; median: 21.4 months) in the low-dose (mostly R0/salvage) group

Dose escalation (79.29 vs. 71.43 Gy EQD-2) was performed in patients with identifiable tumors, defined as positive margins with known localization or detectable via MRI or PET-CT; therefore, 93.6% of high-dose patients had positive margins compared to 4.8% in the lower-dose cohort and, vice versa, 97.3% of margin-positive patients had received high doses. Furthermore, 95.2% of patients with lower-dose radiotherapy had salvage treatment compared to 23.1% of patients with high-dose radiotherapy (*p* < 0.001). Due to these imbalances, it is impossible to distinguish benefits associated with dose escalation (*p* < 0.001 for bPFS and FFBF; Fig. 2a-b) from benefits associated with positive margins [25] or benefits from a negative risk selection in salvage patients (patients in the postoperative setting may not progress despite risk factors, even without treatment [1–3]); accordingly, salvage vs. postoperative radiotherapy was associated with shorter bPFS and FFBF (both *p* < 0.001; Additional file 2: Figure S2a-b). We observed a trend for an improved bPFS (*p* = 0.055) and significantly improved FFBF (*p* = 0.037) in patients with shorter S-RT-Intervals (< 4 months).

Only 13 patients (11%) had received ADT which was associated with a numerical but not significant benefit in bPFS (Additional file 3: Figure S3a-b). Duration of concurrent ADT-usage was heterogeneous in our patient cohort with 6 patients receiving 6 months, which was the minimum duration, a median of 12 months and a maximum of 57.6 months.

### Other factors univariately associated with bPFS or FFBF

Post-surgery, pre-radiotherapy PSA values of ≥0.2 ng/ml were associated with decreased bPFS and FFBF (*p* = 0.022 and *p* = 0.012; Additional file 4: Figure S4a-b; a higher PSA cut-off of 0.5 ng/ml was also associated with bPFS and FFBF: *p* = 0.049 and *p* = 0.022). Elderly patients (≥70 years) did not have a significantly different bPFS (*p* = 0.101) but a trend towards shorter FFBF (*p* = 0.066). Initial, i.e. preoperative PSA values (< 10, 10–20, ≥20 ng/ml) were not associated with bPFS or FFBF after radiotherapy (*p* > 0.1 for all comparisons). Gleason scores of surgical specimens were significantly associated with bPFS (*p* = 0.003) and FFBF (*p* = 0.008; Gleason 8–10 vs. Gleason 6–7; Additional file 4: Figure S4c-d). Roach scores at a cut-off of 15 were not associated with bPFS or FFBF; however, only 21 patients (17.5%) in this advanced patient cohort had Roach scores of < 15. For Roach scores ≥25, there was a significantly decreased bPFS but only a trend for worse FFBF (*p* = 0.043 and *p* = 0.085, respectively; Additional file 4: Figure S4e-f).

### Multivariate models

Multivariate Cox models are shown in Table 2 and Table 3; based on available evidence [5, 6, 19], we decided to enter concurrent ADT-usage into the models despite the insignificant findings in the univariate model which may have been caused by the low number of patients in the ADT-usage group (*n* = 13). Higher Gleason scores were independently associated with shorter and WPRT was independently associated with longer bPFS and FFBF, respectively. High-dose radiotherapy (used in patients with positive margins or detectable disease) showed a trend towards longer bPFS and FFBF (*p* = 0.064 and *p* = 0.073). Significant predictors in Table 2 and Table 3 remained significant when ADT-usage was excluded as a variable and/or Roach scores (cut-off 25) were used instead of or in addition to Gleason scores.

**Table 2:** Multivariate survival model for biochemical progression-free survival (bPFS)

Variable	Hazard Ratio	95% CI	*P*
Elective pelvic radiotherapy (WPRT)	0.484	0.270–0.870	0.015
Detectable tumor and high-dose radiotherapy	0.517	0.257–1.038	0.064
Salvage radiotherapy^c^	1.444	0.704–2.962	0.316
Androgen-deprivation therapy (ADT-) usage^a^	0.764	0.310–1.881	0.558
PSA values at start of radiotherapy above 0.2 ng/ml	1.503	0.850–2.658	0.161
Higher Gleason score of surgical specimens^b^	1.491	1.128–1.973	0.005

^a^Excluding ADT-usage as a variable in a sensitivity model did not change results (i.e. WPRT and Gleason remained significant predictors)

^b^Using the Roach score (cut-off of ≥25) instead of Gleason scores did not change the overall model and did not influence WPRT as a significant predictor

^c^Inclusion of the S-RT-Interval which was not significant in the bPFS univariate model but in the FFBF analysis did not change the overall model and did not influence WPRT as a significant predictor

**Table 3:** Multivariate survival model for freedom from biochemical failure (FFBF); death unrelated to prostate carcinoma was censored and not counted as an event

Variable	Hazard Ratio	95% CI	*P*
Elective pelvic radiotherapy (WPRT)	0.493	0.270–0.898	0.021
Detectable tumor and high-dose radiotherapy	0.525	0.259–1.063	0.073
Salvage radiotherapy^c^	1.547	0.745–3.215	0.242
Androgen-deprivation therapy (ADT-) usage^a^	0.673	0.254–1.786	0.427
PSA values at start of radiotherapy above 0.2 ng/ml	1.631	0.904–2.943	0.104
Higher Gleason score of surgical specimens^b^	1.441	1.080–1.922	0.013

^a^Excluding ADT-usage as a variable in a sensitivity model did not change results (i.e. WPRT and Gleason remained significant predictors)

^b^Using the Roach score (cut-off of ≥25) instead of Gleason scores did not change the overall model and did not influence WPRT as a significant predictor

^c^Inclusion of the S-RT-Interval which was not significant in the bPFS univariate model but in the FFBF analysis did not change the overall model and did not influence WPRT as a significant predictor

### Salvage and postoperative subgroups with or without detectable disease

To analyze if the association of WPRT with FFBF and bPFS observed in the whole cohort was present in both the salvage and postoperative (including adjuvant) setting, we performed subgroup analyses: WPRT was significantly associated with FFBF and bPFS in the salvage setting (*p* = 0.029 for bPFS and *p* = 0.039 for FFBF; Additional file 5: Figure S5a-b) but there was no difference when radiotherapy was applied postoperatively without confirmed rise in PSA (*p* = 0.616 for bPFS and *p* = 0.56 for FFBF; Additional file 5: Figure S5c-d). However, this analysis is confounded by our treatment approach: Most patients (96.8%) treated postoperatively had positive margins and patients with positive margins had dose escalation in almost all cases (97.3%); vice versa, patients treated in the salvage setting had mostly negative margins (74.1%) and the lower-dose approach (69%) for undetectable disease and negative margins. Therefore, we cannot specify if WPRT was associated with FFBF and bPFS in this subgroup because of the salvage situation, mostly undetectable tumor localizations, lower doses to the prostate bed or because of a combination of these factors. Because of the lower numbers in the subgroups and the significant overlap of variables, a multivariate model was not applicable in the subgroups.

### Toxicity

Grade ≥ 2 toxicity occurred in 20 patients (16.7%) with an increased risk in patients who received WPRT compared to PBRT (*p* = 0.005) and in patients who had 79.29 Gy compared to 71.43 Gy to the prostatic bed (*p* = 0.043). Patients who received low-dose radiotherapy without pelvic irradiation had the lowest grade 2+ toxicity risk (3.4%) followed by high-dose PBRT (12.5%), low-dose WPRT (15.4%) and high-dose WPRT (36.7%). There was no grade 3 gastrointestinal toxicity and there were 10 cases of grade 3 genitourinary toxicity, five after WPRT/PBRT (11.6%) and five after PBRT-only (6.5%); all patients with grade 3 events had received 79.29 Gy; incontinence at baseline which worsened after irradiation counted as an event.

### Supplementary analysis including patients with localized or node-positive disease

All details for the pooled patient cohort of node-negative patients which included patients with localized disease (49/52 high-risk, 3/52 intermediate risk) and the full cohort, including patients with node-positive disease (*n* = 40; all M0) is shown in Additional file 7: Table S1 and Additional file 6: Figure S6. Briefly, WPRT was associated with better bPFS and FFBF in the pooled cohorts as well but it is unknown if results can be extrapolated to any subgroup other than locally advanced node-negative patients.

## Discussion

With this study, we provide a retrospective analysis on the addition of WPRT to dose-escalated PBRT after prostatectomy for locally advanced node-negative tumors. Most available studies reported on patients treated with prostate bed doses ranging from 61.2–72 Gy [19, 26–30] with only two European series reporting on toxicity of higher-dose series (EQD-2: 75–77 Gy) [15, 31]. WPRT doses in recent studies ranged from 40 to 54 Gy. WPRT was associated with a bPFS benefit in some studies [26, 29, 30] while others described an association limited to subgroups [28] or found no association at all [27, 32]. The strongest but not yet fully published evidence so far in support of combining PBRT and WPRT comes from an interim analysis of the RTOG-0534/SPPORT trial which showed significantly different 5-year failure-free survival rates between PBRT, PBRT plus ADT and WPRT plus ADT arms of 71.7, 82.7 and 89.1%, respectively. Additionally, a trend towards increased metastases-free survival was reported when WPRT plus ADT was compared to the PBRT arm without ADT (*p* = 0.014; the 3-arm trial requires a *P* value of 0.0125 for one-sided significance [19]). The trial protocol allowed for doses between 64.8–70.2 Gy and (dose-)subgroup analyses have not yet been reported. Biochemical failure rates were calculated using the Phoenix definition (PSA 2 ng/ml above nadir or clinical progression); patients who initiated second salvage prior to this definition were censored. In our analysis, we used a definition of two PSA rises and counted patients who initiated second salvage as events, even if salvage was initiated after one increase in PSA levels. Our dose escalation strategy does not allow for a quantification of the benefit associated with 79.29 Gy EQD-2 compared to 71.43 Gy EQD-2 because we cannot separate benefits associated with positive margins [25] from benefits of dose escalation which was almost exclusively used in margin-positive patients plus a minority (6.4%) who received escalation after detection of recurrence in imaging (MRI as published [33] or PET-CT). Additionally, patients who received the higher dose were mostly treated postoperatively (76.9%) while 95.2% of patients with lower-dose treatment had a salvage strategy associated with a more unfavorable risk selection. Therefore, we did not attempt to quantify benefits of the two dose escalation levels but only analyzed benefits of WPRT in combination with PBRT at the high-dose-end of the currently used spectrum, the use of which was more evenly distributed across subgroups with fewer baseline imbalances as detailed in Table 1.

We found a 5-year bPFS benefit of 12.1% (FFBF: 11.6%) after WPRT/PBRT compared to PBRT-only which is higher compared to the 6.4% benefit observed in the RTOG-0534 dataset after WPRT and ADT vs. PBRT and ADT.

However, due to baseline imbalances and the retrospective nature of our analysis, the univariate model has to be interpreted with caution. Nevertheless, multivariate models showed a significant association of WPRT with longer bPFS and FFBF and thus, for the analyzed cohort, confirms the preliminary results of RTOG-0534. This was also true for the pooled patient cohort which includes patients who did not have advanced disease and the full cohort which also included node-positive patients (T1–4/N0–1; all M0); however, as mentioned it is uncertain if the associations of WPRT with outcomes can be extrapolated to any subgroup other than locally advanced, node-negative patients in our cohort because almost all patients with positive nodes were treated with WPRT while only 8 patients with T1–2/N0 tumors had WPRT.

Interestingly, the association of WPRT with longer bPFS in our cohort was consistent in the subgroup of patients who had salvage radiotherapy but not in patients with postoperative/adjuvant strategy. We cannot determine if the benefit of WPRT occurred because of a worse risk selection due to the salvage situation, the negative margin situation or the lower dose approach, as these factors overlapped. Likely, patients with negative margins, negative imaging and rising PSA values will have a higher risk of (undetected) microscopic disease in the lymph nodes compared to patients with an identifiable lesion (including R1) which explains rising or stable PSA values. Based on our dataset, the benefit of larger fields may be smaller in patients with positive margins. This hypothesis is in line with the previously reported improved outcomes for patients with positive margins in the adjuvant settings using PBRT [34, 35] and in the nomogram published by Tendulkar et al. for the salvage [25] situation (83% of patients had PBRT). In patients with rising PSA values and an identifiable lesion, the risk of an occult (second) lesion may be lower compared to the risk in patients without any identified lesion explaining rising PSA values. However, this hypothesis cannot be ascertained with our dataset and we look forward to subgroup analysis of the RTOG-0534 trial which allowed for patients with positive or negative margins.

WPRT and dose escalation in our dataset were associated with a significantly increased risk of toxicity; furthermore, pelvic radiotherapy increased symptom burden and decision regret for radiotherapy (details will be reported separately). A higher risk of side effects has been described for dose-escalated radiotherapy in this setting even if most advanced techniques are applied [13–15]; furthermore, an increased incidence of toxicity following WPRT in line with our data has been reported previously [15]. For this reason, improved accuracy in the identification of patients who benefit from WPRT is mandatory.

Our study has several weaknesses; first, it is a retrospective analysis and therefore hypothesis-generating; second, it is uncertain if our data can be extrapolated to patients who received radiation doses below 70 Gy; third, ADT-usage was infrequent and duration was heterogeneous. Finally, we did not perform a patterns-of-recurrence analysis and did not systematically analyze imaging prior to inclusion; therefore, we do not know how many recurrences occurred in the pelvis and we cannot provide details on patients with selective dose escalation to lymph nodes after advanced imaging as they were excluded from the dataset. Despite these shortcomings our results indicate that WPRT was associated with longer bPFS and FFBF in patients with locally advanced node-negative prostate carcinoma who received radiotherapy to the prostatic bed after prostatectomy; although the result was significant in the whole patient group, subgroup analyses indicated that the benefit was more pronounced in patients with rising PSA values without detectable disease or positive margins. Our data together with the final results of RTOG-0534 will help to further refine patient selection in the postoperative/salvage setting.

## Conclusions

We found a significant benefit in bPFS associated with WPRT in patients with locally advanced prostate carcinoma who underwent dose-escalated radiotherapy at the prostate bed. Incidence of grade 2+ toxicity was higher in patients who had received WPRT or higher doses (79.29 Gy compared to 71.43 Gy). In subset analyses, the association of WPRT with longer bPFS was only observed in patients with rising PSA values but not in patients with non-salvage postoperative radiotherapy for positive margins. Taken together, our data indicate that there is a benefit of WPRT vs. PBRT in patients with rising PSA values.

## Additional files

Additional file 1: **Figure S1.** CONSORT-like diagram, illustrating selection criteria for this retrospective analysis. (DOC 47 kb)

Additional file 2: **Figure S2.** (a-b) Univariate survival analyses showing postoperatively treated patients compared to patients with rising PSA values (salvage treatment). (DOCX 181 kb)

Additional file 3: **Figure S3.** (a-b) Univariate survival analyses of patients treated with or without concurrent ADT. (DOCX 178 kb)

Additional file 4: **Figure S4.** (a-b) Univariate survival analyses of patients with PSA values of ≥0.2 ng/ml compared to lower values. (c-d) Univariate survival analyses of patients with Gleason scores of 8-10 compared to Gleason 6-7 (surgical specimen). (e-f) Univariate survival analyses of patients with Roach scores of ≥25 compared to lower scores. (DOCX 547 kb)

Additional file 5: **Figure S5.** (a-b) Univariate survival analyses of patients treated with WPRT and PBRT compared to PBRT only in the subgroup of patients with rising PSA values (salvage cohort). (c-d) Univariate survival analyses of patients treated with WPRT and PBRT compared to PBRT only in the subgroup of patients treated postoperatively without salvage indication. (DOCX 288 kb)

Additional file 6: **Figure S6.** Univariate survival analyses comparing FFBF and bPFS between the analysed cohort of patients with locally advanced tumors and patients with localized tumors and node-positive tumors at our department. (DOCX 373 kb)

Additional file 7: **Table S1.** This file shows multivariate models which include patients with localized disease and/or patients with node-positive tumors. (DOC 76 kb)

## Data Availability

The dataset generated and analyzed during the current study are available from the corresponding author on reasonable request.

## Abbreviations

ADT: Androgen depression therapy
bPFS: biochemical progression-free survival
CBCT: Cone-beam computed tomography
CI: Confidence interval
CTV: Clinical target volume
EQD-2: Biologically-equivalent dose (in fractions of 2 Gy)
FFBF: Freedom from biochemical failure
HR: Hazard ratio
IGRT: Image-guided radiation therapy
IMRT: Intensity-modulated radiation therapy
MRI: Magnetic resonance imaging
OS: Overall survival
PBRT: Radiotherapy of the prostatic fossa (without WPRT; fossa-only)
PSA: Prostate-specific antigen
PTV: Planning target volume
SIB: Simultaneous integrated boost
S-RT-Interval: Interval between surgery and start of radiation therapy
VMAT: Volumetric Modulated Arc Therapy
WPRT: Elective pelvic nodal irradiation (in addition to PBRT)
