# Supplementary material for: Postoperative elective pelvic nodal irradiation compared to prostate bed irradiation in locally advanced prostate cancer – a retrospective analysis of dose-escalated patients
Source: Radiat Oncol. 2019 Jun 7;14:96. doi: 10.1186/s13014-019-1301-5 (PMC6554899; doi:10.1186/s13014-019-1301-5)
Supplement: Supplementary file 1 — Figure S1. CONSORT-like diagram, illustrating selection criteria for this retrospective analysis. (DOC 47 kb) [file 13014_2019_1301_MOESM1_ESM.doc]

Assessed for eligibility (n=244)

Excluded from analysis (n=32)

- No radical prostatectomy but palliative surgery
- Not irradiation to the prostatic fossa (lymph node radiotherapy, only)
- Concurrent stereotactic radiotherapy to lymph nodes
- Concurrent M1-disease
- One patient retracted his consent for any data usage including retrospective survival data
- Selective dose escalation to the bladder wall
- Prior pelvic radiotherapy (testicular cancer)
- Two Patients had a higher doses to the pelvis (50 Gy)^§^

Patients with pathologically positive lymph nodes (pN1); n=40; of whom 38 had received WPRT (95% vs. n=2, 5% PBRT)

Patients with localized disease (T1-2 N0): n=52 of whom 8 had received WPRT (15.4% vs. n=44, 84.6% PBRT)

212 patients

§ One patient had a slightly modified fractionation concept: 44 Gy to the pelvis plus PTV-1/2 doses of 27.04 Gy and 30.81 Gy; this concept results in a similar EQD-2 for PTV-1/2 compared to the standard concept; therefore, this patient was not excluded: PTV-1 and -2 EQD-2: 71.66 and 78.61 Gy (standard concept: 71.43 and 79.29 Gy); this patient was regularly analyzed in the pelvis high-dose group.

Patients with locally advanced tumors T3‑4 N0: n=120 of whom 43 had received WPRT

Supplementary Figure

S-1

**Primary analysis**

*Reported as supplementary data*

*Reported as supplementary data*
