# Supplementary material for: Postoperative elective pelvic nodal irradiation compared to prostate bed irradiation in locally advanced prostate cancer – a retrospective analysis of dose-escalated patients
Source: Radiat Oncol. 2019 Jun 7;14:96. doi: 10.1186/s13014-019-1301-5 (PMC6554899; doi:10.1186/s13014-019-1301-5)
Supplement: Supplementary file 2 — Figure S2. (a-b) Univariate survival analyses showing postoperatively treated patients compared to patients with rising PSA values (salvage treatment). (DOCX 181 kb) [file 13014_2019_1301_MOESM2_ESM.docx]

**Supplementary figure S-2**

a)

**Biochemical progression-free survival (bPFS) for postoperative (0) compared to salvage (1) radiotherapy indication (RT_Indication)**

**Months**

**bPFS**


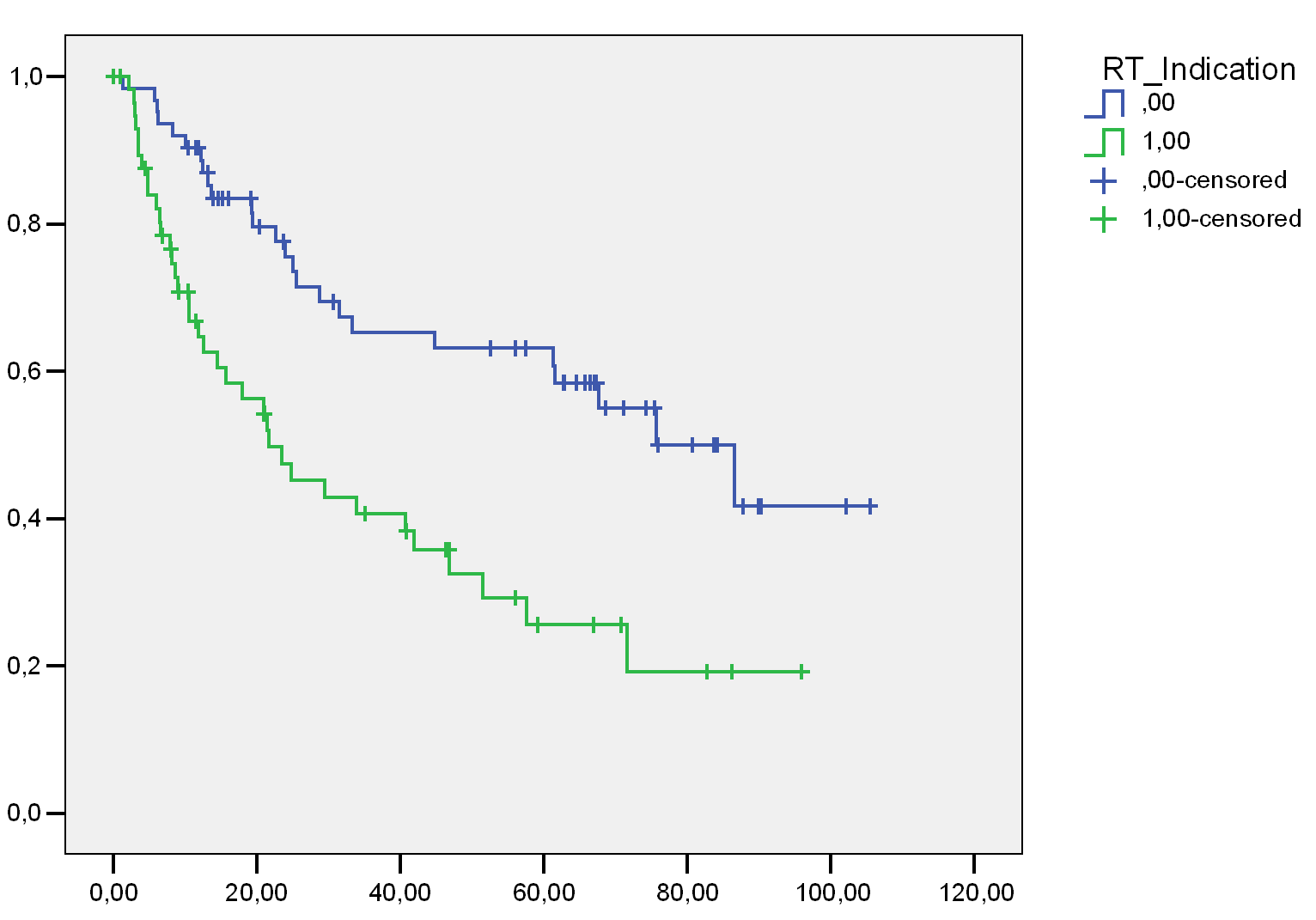


| **No. at risk** |  |  |  |  |  |  |  |
| --- | --- | --- | --- | --- | --- | --- | --- |
| **Months** | **0** | **20** | **40** | **60** | **80** | **100** | **120** |
| **Postoperative*** | 62 | 41 | 31 | 26 | 9 | 2 | 0 |
| **Salvage** | 58 | 27 | 17 | 6 | 3 | 0 | 0 |

* Postoperative treatment indication included patients with positive and with negative margins (adjuvant)

RT_Indication: 0=Postoperative prior to confirmed rising PSA or adjuvant prior to rising PSA; 1=Salvage, i.e. confirmed PSA increase

b)

**Freedom from biochemical failure (FFBF) for postoperative (0) compared to salvage (1) radiotherapy indication (RT_Indication)**

**Months**

**FFBF**


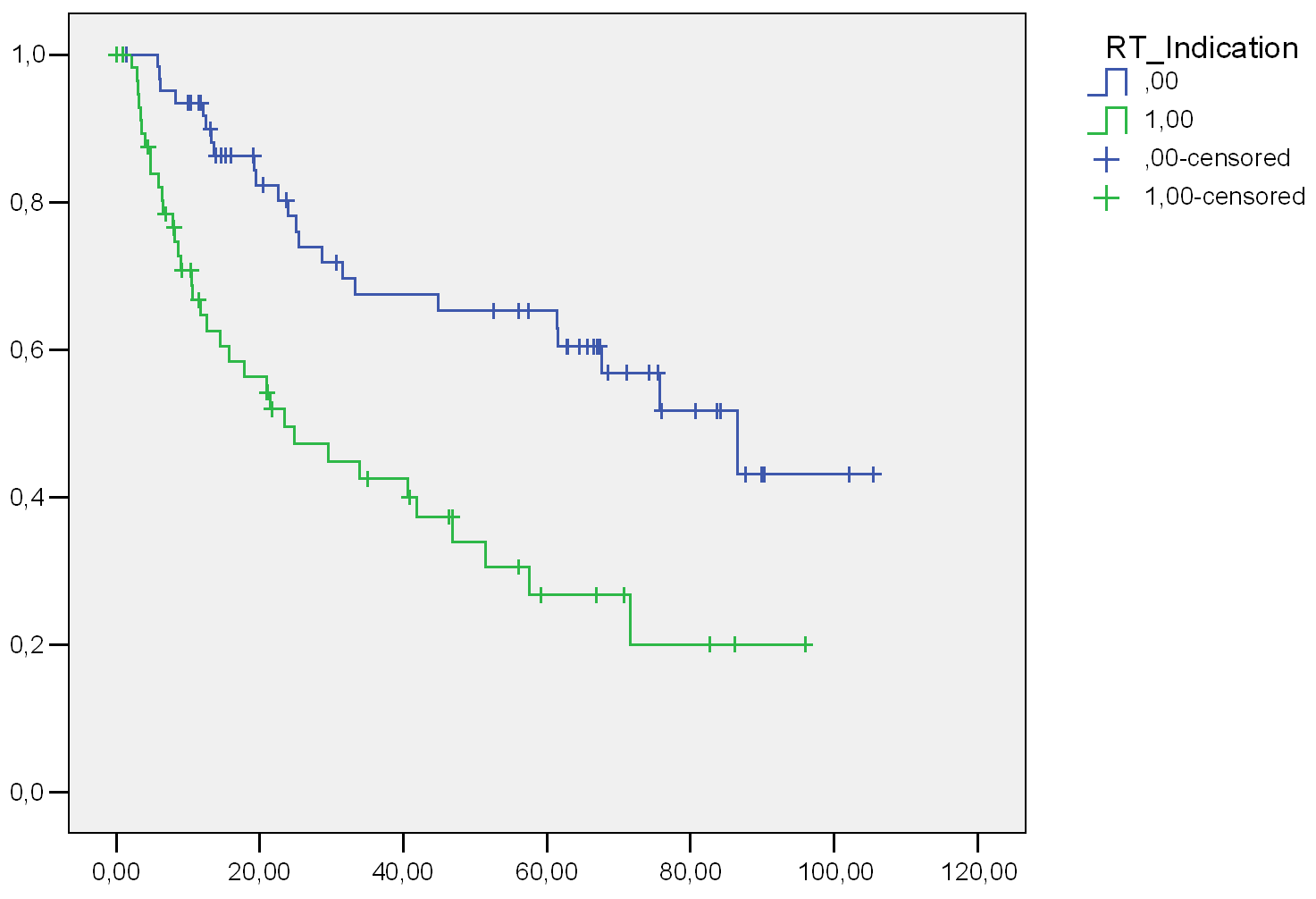


| **No. at risk** |  |  |  |  |  |  |  |
| --- | --- | --- | --- | --- | --- | --- | --- |
| **Months** | **0** | **20** | **40** | **60** | **80** | **100** | **120** |
| **Postoperative*** | 62 | 41 | 31 | 26 | 9 | 2 | 0 |
| **Salvage** | 58 | 27 | 17 | 6 | 3 | 0 | 0 |

* Postoperative treatment indication included patients with positive and with negative margins (adjuvant)

RT_Indication: 0=Postoperative prior to confirmed rising PSA or adjuvant prior to rising PSA; 1=Salvage, i.e. confirmed PSA
