# Supplementary material for: Postoperative elective pelvic nodal irradiation compared to prostate bed irradiation in locally advanced prostate cancer – a retrospective analysis of dose-escalated patients
Source: Radiat Oncol. 2019 Jun 7;14:96. doi: 10.1186/s13014-019-1301-5 (PMC6554899; doi:10.1186/s13014-019-1301-5)
Supplement: Supplementary file 3 — Figure S3. (a-b) Univariate survival analyses of patients treated with or without concurrent ADT. (DOCX 178 kb) [file 13014_2019_1301_MOESM3_ESM.docx]

**Supplementary figure S-3**

a)

**Biochemical progression-free survival (bPFS) for patients with (1) or without (0) concurrent androgen deprivation therapy (Concurrent_ADT)**

**Months**

**bPFS**


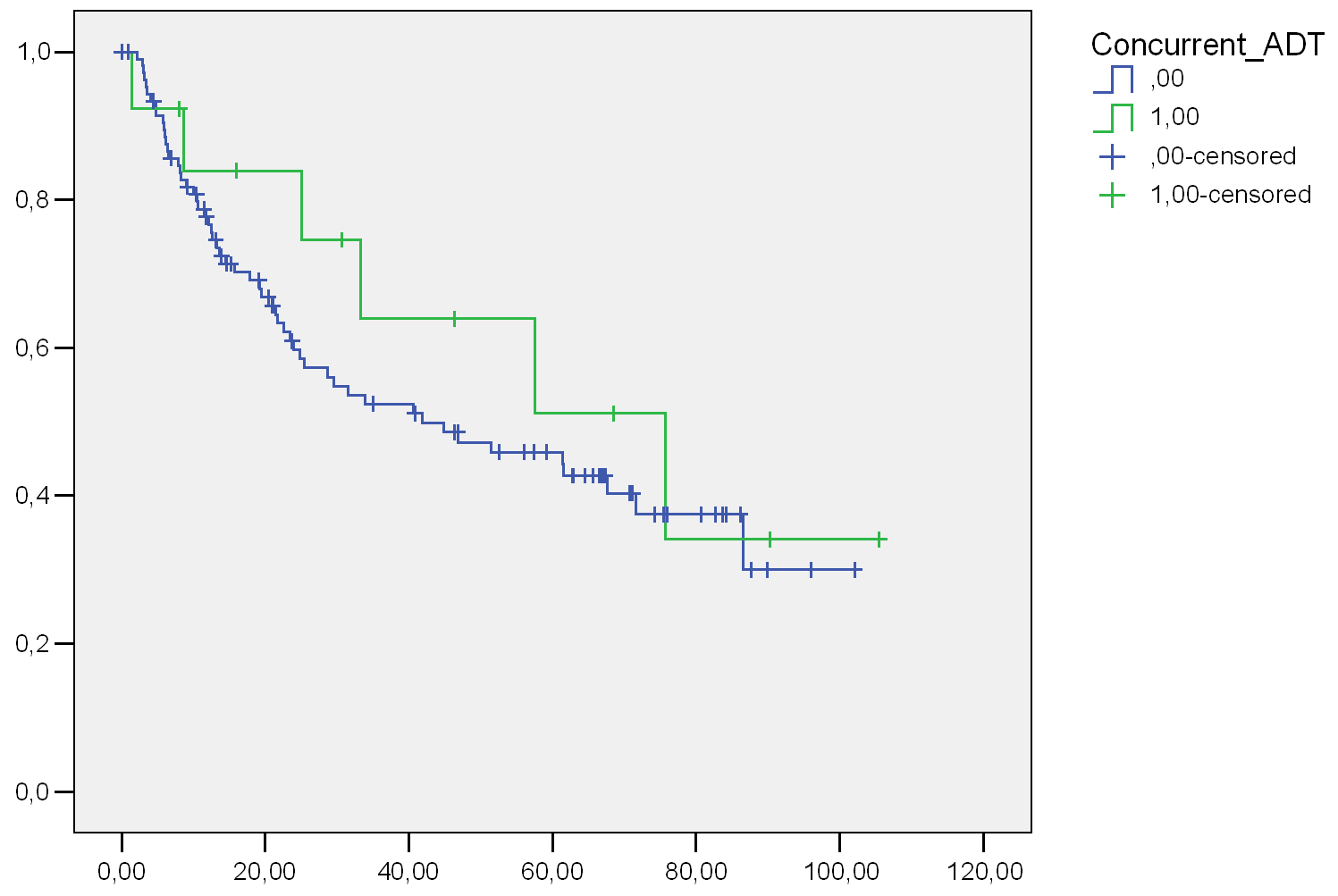


| **No. at risk** |  |  |  |  |  |  |  |
| --- | --- | --- | --- | --- | --- | --- | --- |
| **Months** | **0** | **20** | **40** | **60** | **80** | **100** | **120** |
| **Concurrent ADT** | 13 | 9 | 6 | 4 | 2 | 1 | 0 |
| **No concurrent ADT** | 107 | 59 | 42 | 29 | 10 | 1 | 0 |

Concurrent_ADT: 0=Patient did not have concurrent ADT; 1=Patient had concurrent ADT

b)


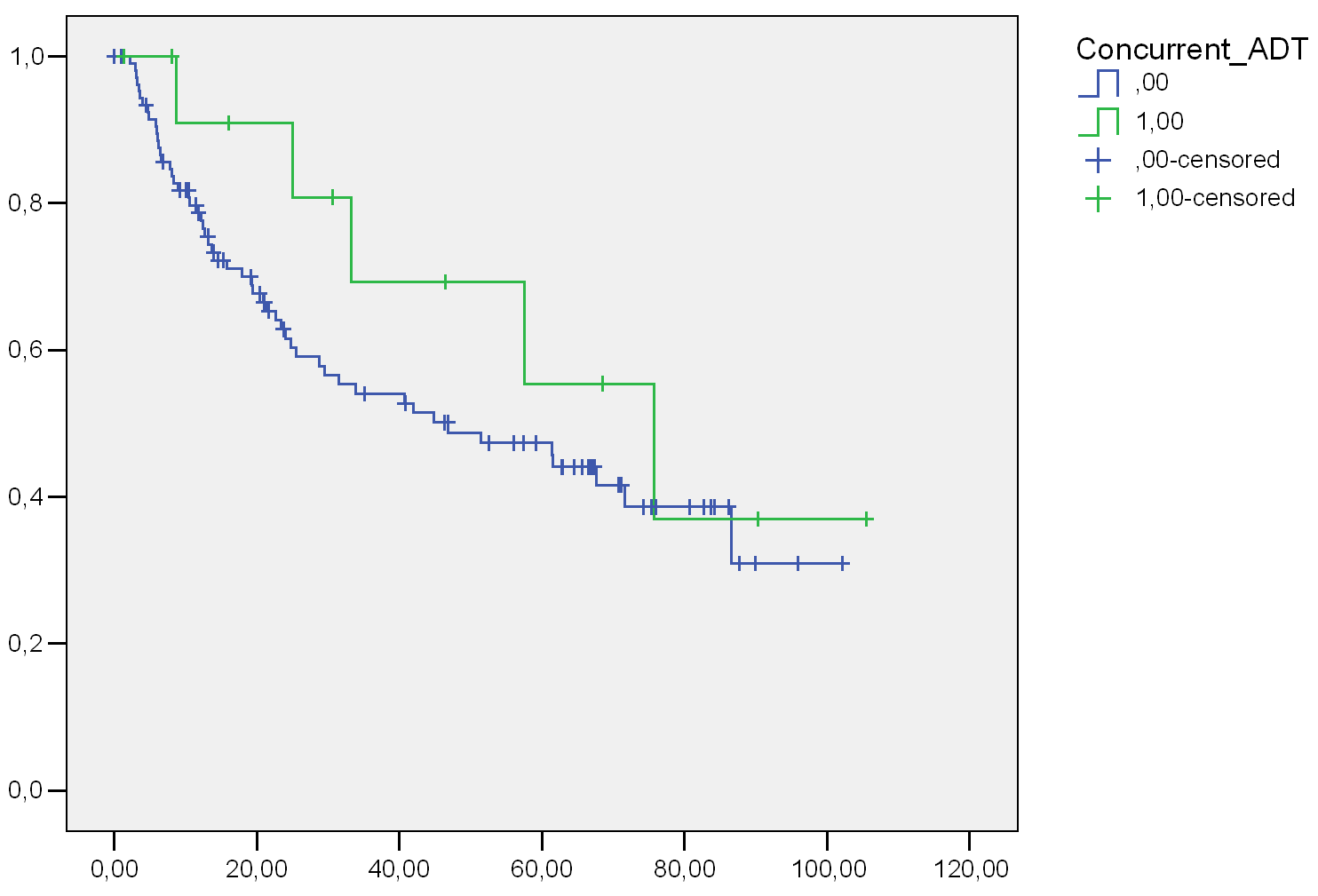


**Freedom from biochemical failure (FFBF) for patients with (1) or without (0) concurrent androgen deprivation therapy (Concurrent_ADT)**

**Months**

**FFBF**

| **No. at risk** |  |  |  |  |  |  |  |
| --- | --- | --- | --- | --- | --- | --- | --- |
| **Months** | **0** | **20** | **40** | **60** | **80** | **100** | **120** |
| **Concurrent ADT** | 13 | 9 | 6 | 4 | 2 | 1 | 0 |
| **No concurrent ADT** | 107 | 59 | 42 | 29 | 10 | 1 | 0 |

Concurrent_ADT: 0=Patient did not have concurrent ADT; 1=Patient had concurrent ADT
