# Supplementary material for: Postoperative elective pelvic nodal irradiation compared to prostate bed irradiation in locally advanced prostate cancer – a retrospective analysis of dose-escalated patients
Source: Radiat Oncol. 2019 Jun 7;14:96. doi: 10.1186/s13014-019-1301-5 (PMC6554899; doi:10.1186/s13014-019-1301-5)
Supplement: Supplementary file 4 — Figure S4. (a-b) Univariate survival analyses of patients with PSA values of ≥0.2 ng/ml compared to lower values. (c-d) Univariate survival analyses of patients with Gleason scores of 8-10 compared to Gleason 6-7 (surgical specimen). (e-f) Univariate survival analyses of patients with Roach scores of ≥25 compared to lower scores. (DOCX 547 kb) [file 13014_2019_1301_MOESM4_ESM.docx]

**Supplementary figure S-4**

a)

**Biochemical progression-free survival (bPFS) in patients with pre-radiotherapy PSA values above or below the cut-off (0.2 ng/ml)**

**Months**

**bPFS**


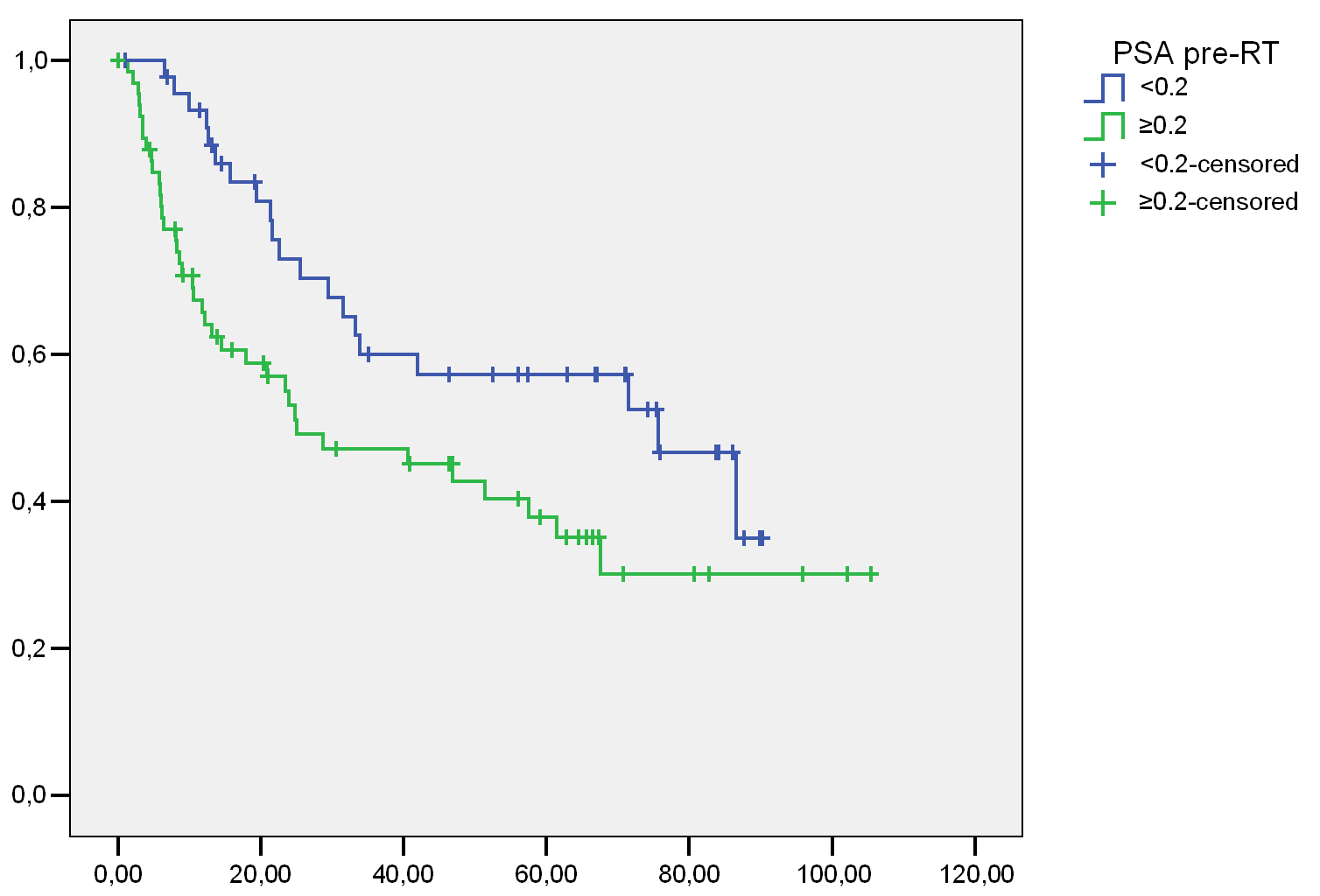


| **No. at risk** |  |  |  |  |  |  |  |
| --- | --- | --- | --- | --- | --- | --- | --- |
| **Months** | **0** | **20** | **40** | **60** | **80** | **100** | **120** |
| **Pre-RT PSA <0.2 ng/ml (0)** | 46 | 31 | 22 | 17 | 7 | 0 | 0 |
| **Pre-RT PSA ≥0.2 ng/ml (1)** | 67 | 33 | 23 | 14 | 5 | 2 | 0 |

PSApreRT02: 0=PSA prior to radiotherapy <0.2; 1=PSA prior to radiotherapy ≥0.2

b)

**Freedom from biochemical failure (FFBF) in patients with pre-radiotherapy PSA values above or below the cut-off (0.2 ng/ml)**

**Months**

**FFBF**


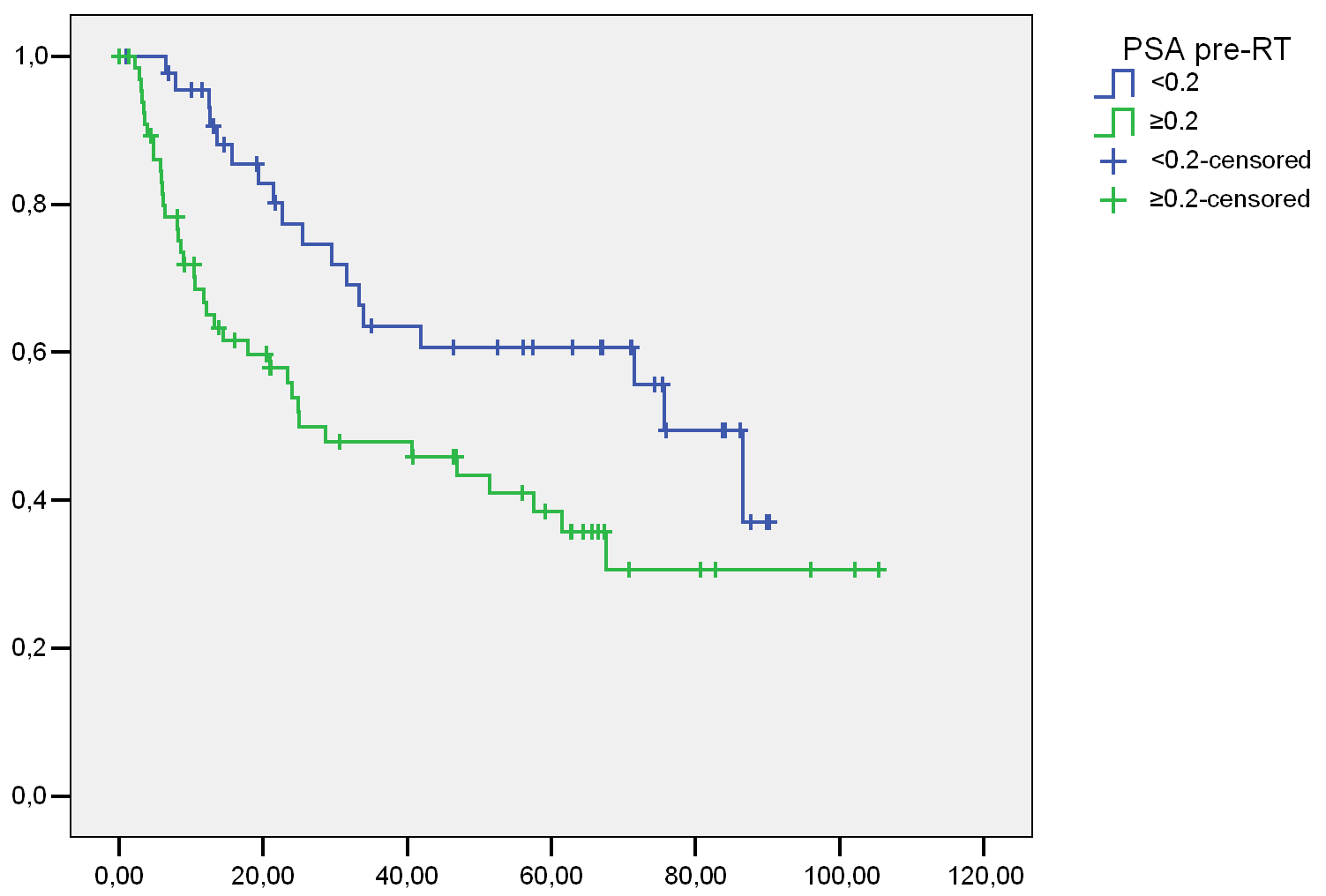


| **No. at risk** |  |  |  |  |  |  |  |
| --- | --- | --- | --- | --- | --- | --- | --- |
| **Months** | **0** | **20** | **40** | **60** | **80** | **100** | **120** |
| **Pre-RT PSA <0.2 ng/ml (0)** | 46 | 31 | 22 | 17 | 7 | 0 | 0 |
| **Pre-RT PSA ≥0.2 ng/ml (1)** | 67 | 33 | 23 | 14 | 5 | 2 | 0 |

PSApreRT02: 0=PSA prior to radiotherapy <0.2; 1=PSA prior to radiotherapy ≥0.2

c)


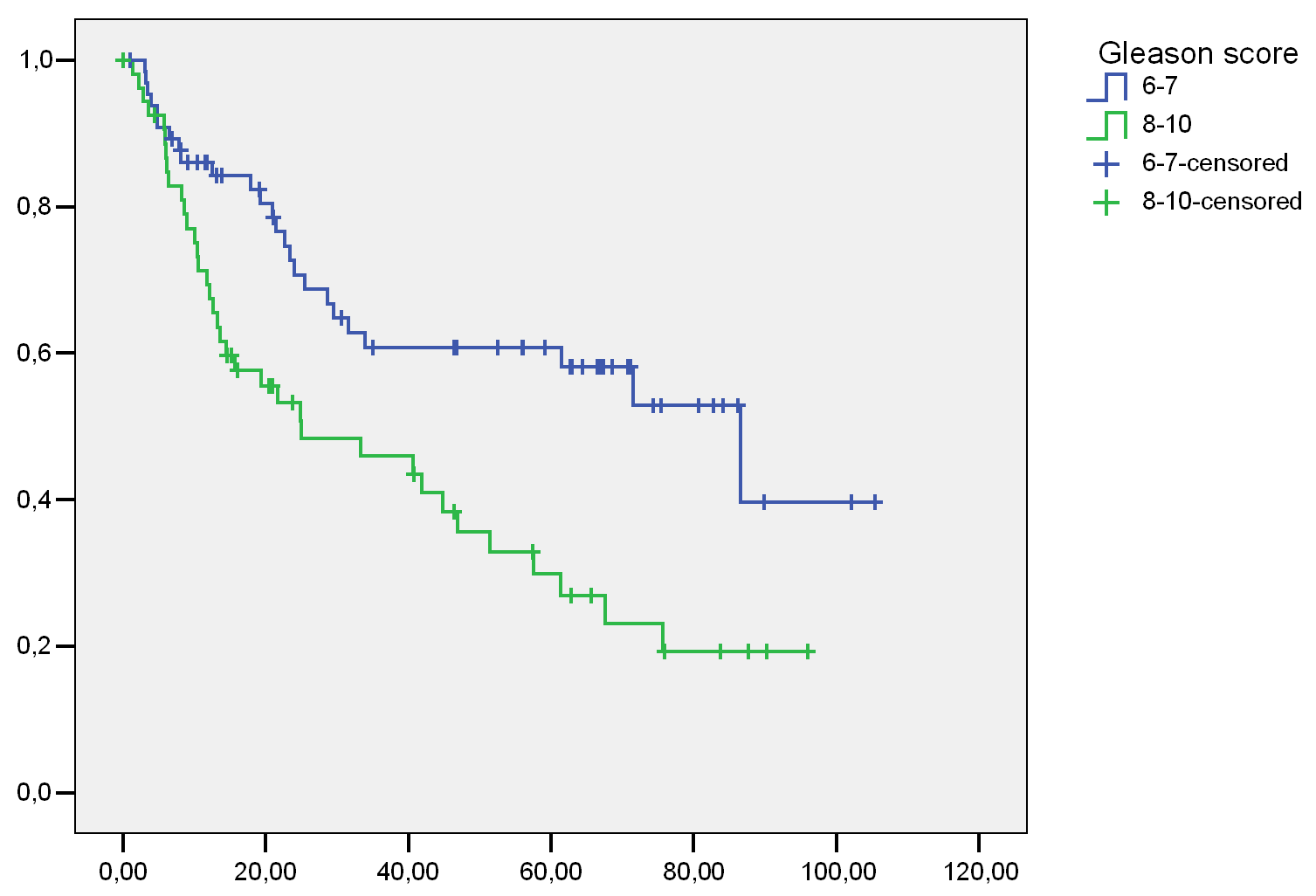


**Biochemical progression-free survival (bPFS) in patients with Gleason scores of 6-7 compared to 8-10**

**Months**

**bPFS**

| **No. at risk** |  |  |  |  |  |  |  |
| --- | --- | --- | --- | --- | --- | --- | --- |
| **Months** | **0** | **20** | **40** | **60** | **80** | **100** | **120** |
| **Gleason 6-7** | 66 | 42 | 29 | 23 | 8 | 2 | 0 |
| **Gleason 8-10** | 54 | 26 | 19 | 10 | 4 | 0 | 0 |

Gleason_Surgery: 0=Gleason score of surgical specimen 6-7; 1=Gleason score of surgical specimen 8-10

d)


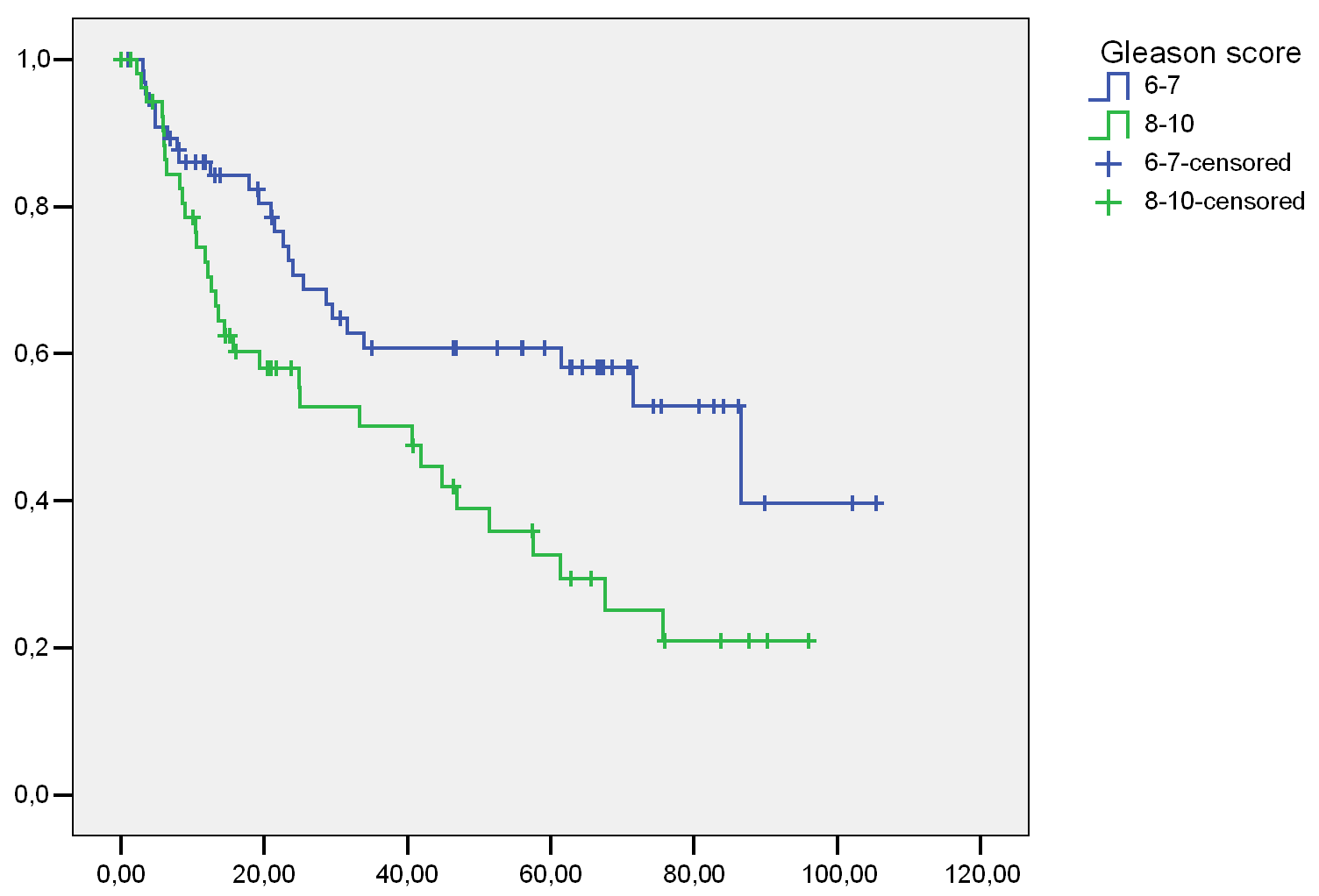


**Freedom from biochemical failure (FFBF) in patients with Gleason scores of 6-7 compared to 8-10**

**Months**

**FFBF**

| **No. at risk** |  |  |  |  |  |  |  |
| --- | --- | --- | --- | --- | --- | --- | --- |
| **Months** | **0** | **20** | **40** | **60** | **80** | **100** | **120** |
| **Gleason 6-7** | 66 | 42 | 29 | 23 | 8 | 2 | 0 |
| **Gleason 8-10** | 54 | 26 | 19 | 10 | 4 | 0 | 0 |

Gleason_Surgery: 0=Gleason score of surgical specimen 6-7; 1=Gleason score of surgical specimen 8-10

e)


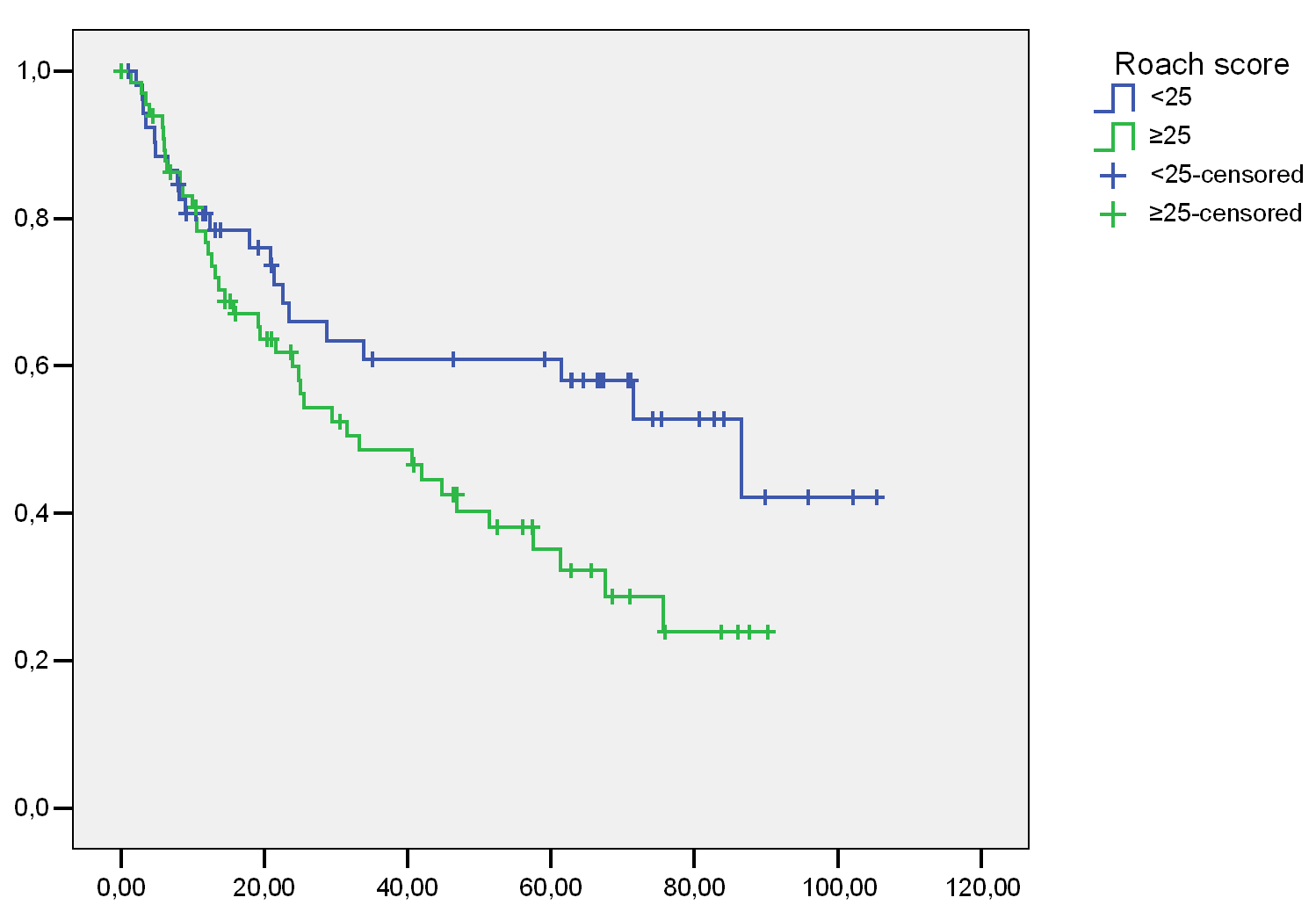


**Biochemical progression-free survival (bPFS) in patients with Roach scores of <25 compared to ≥25**

**Months**

**bPFS**

| **No. at risk** |  |  |  |  |  |  |  |
| --- | --- | --- | --- | --- | --- | --- | --- |
| **Months** | **0** | **20** | **40** | **60** | **80** | **100** | **120** |
| **Roach score <25** | 53 | 31 | 23 | 21 | 8 | 2 | 0 |
| **Roach score** **≥25** | 67 | 37 | 25 | 12 | 4 | 0 | 0 |

Roach_25: 0=Roach score <25; 1=Roach score ≥25

f)


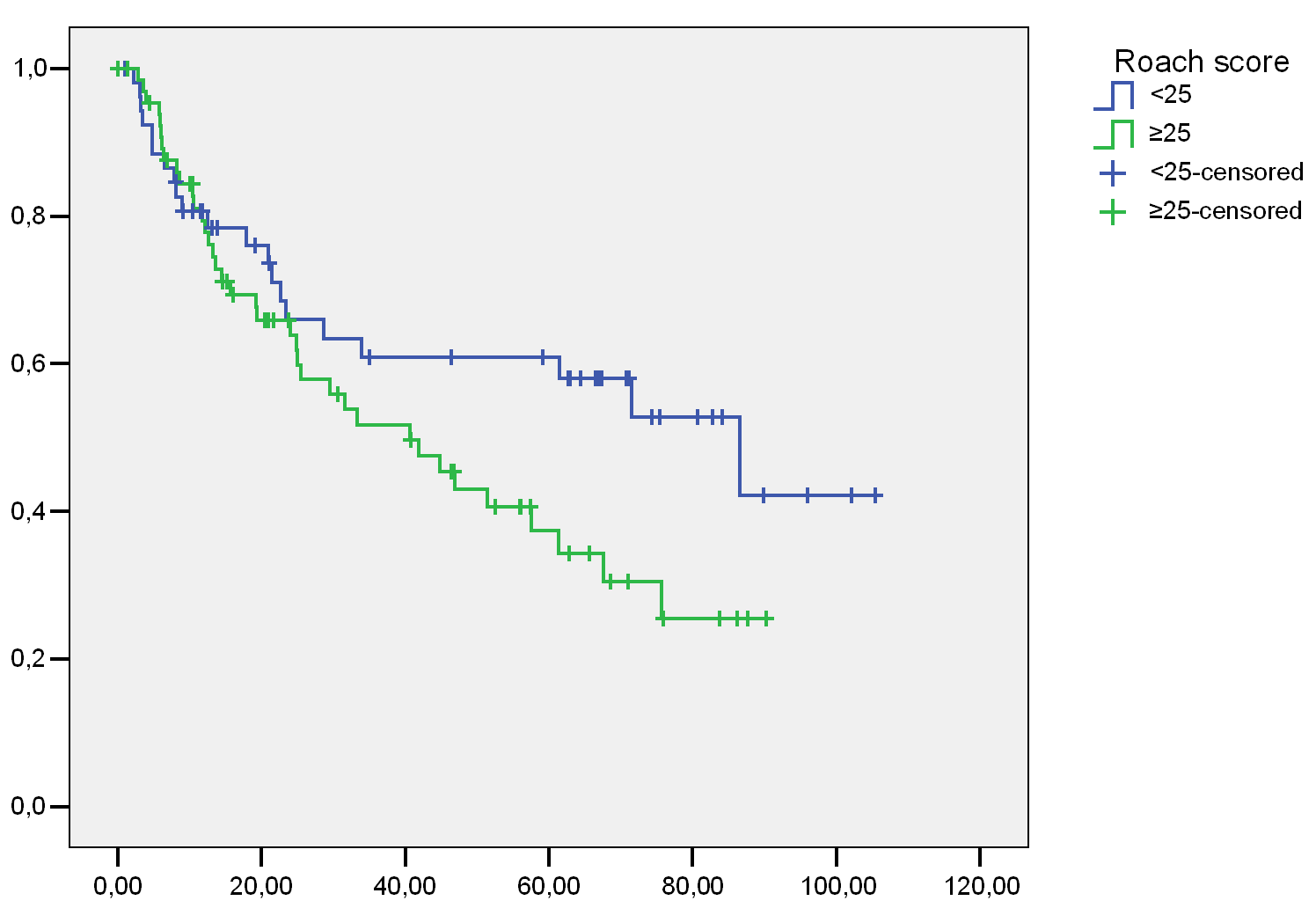


**Freedom from biochemical failure (FFBF) in patients with Roach scores of <25 compared to ≥25**

**Months**

**FFBF**

| **No. at risk** |  |  |  |  |  |  |  |
| --- | --- | --- | --- | --- | --- | --- | --- |
| **Months** | **0** | **20** | **40** | **60** | **80** | **100** | **120** |
| **Roach score <25** | 53 | 31 | 23 | 21 | 8 | 2 | 0 |
| **Roach score ≥25** | 67 | 37 | 25 | 12 | 4 | 0 | 0 |

Roach_25: 0=Roach score <25; 1=Roach score ≥25
