# Supplementary material for: Postoperative elective pelvic nodal irradiation compared to prostate bed irradiation in locally advanced prostate cancer – a retrospective analysis of dose-escalated patients
Source: Radiat Oncol. 2019 Jun 7;14:96. doi: 10.1186/s13014-019-1301-5 (PMC6554899; doi:10.1186/s13014-019-1301-5)
Supplement: Supplementary file 5 — Figure S5. (a-b) Univariate survival analyses of patients treated with WPRT and PBRT compared to PBRT only in the subgroup of patients with rising PSA values (salvage cohort). (c-d) Univariate survival analyses of patients treated with WPRT and PBRT compared to PBRT only in the subgroup of patients treated postoperatively without salvage indication. (DOCX 288 kb) [file 13014_2019_1301_MOESM5_ESM.docx]

**Supplementary figure S-5**

a)


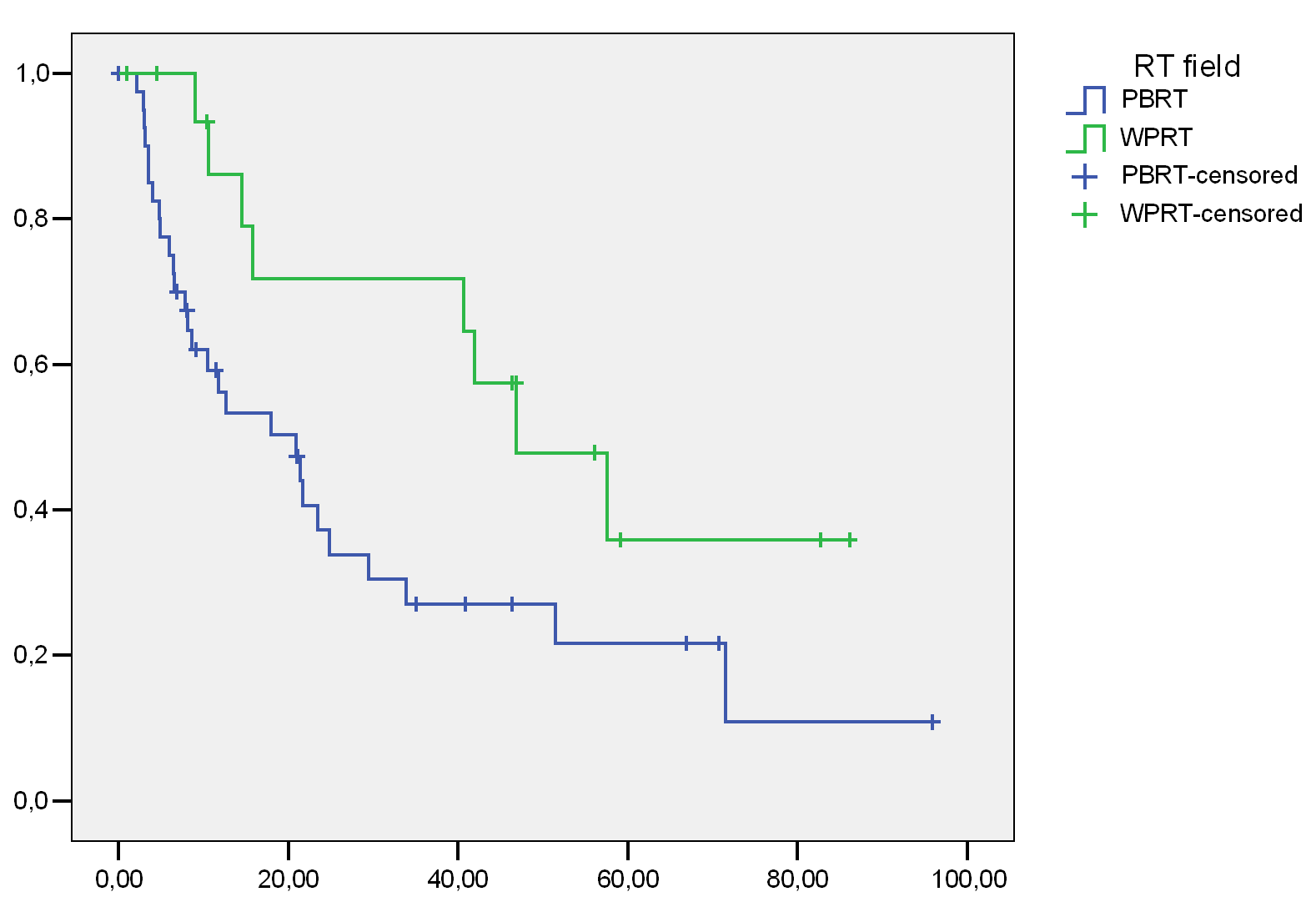


**Biochemical progression-free survival (bPFS) in patients, who had salvage whole pelvis radiotherapy (WPRT) compared to salvage fossa-only radiotherapy (PBRT)**

**Months**

**bPFS**

| **No. at risk** |  |  |  |  |  |  |  |
| --- | --- | --- | --- | --- | --- | --- | --- |
| **Months** | **0** | **20** | **40** | **60** | **80** | **100** | **120** |
| **Whole pelvis (WPRT)** | 17 | 10 | 10 | 2 | 2 | 0 | 0 |
| **Fossa-only (PBRT)** | 41 | 17 | 7 | 4 | 1 | 0 | 0 |

Pelvic_RT: 0=PBRT; 1=WPRT

b)


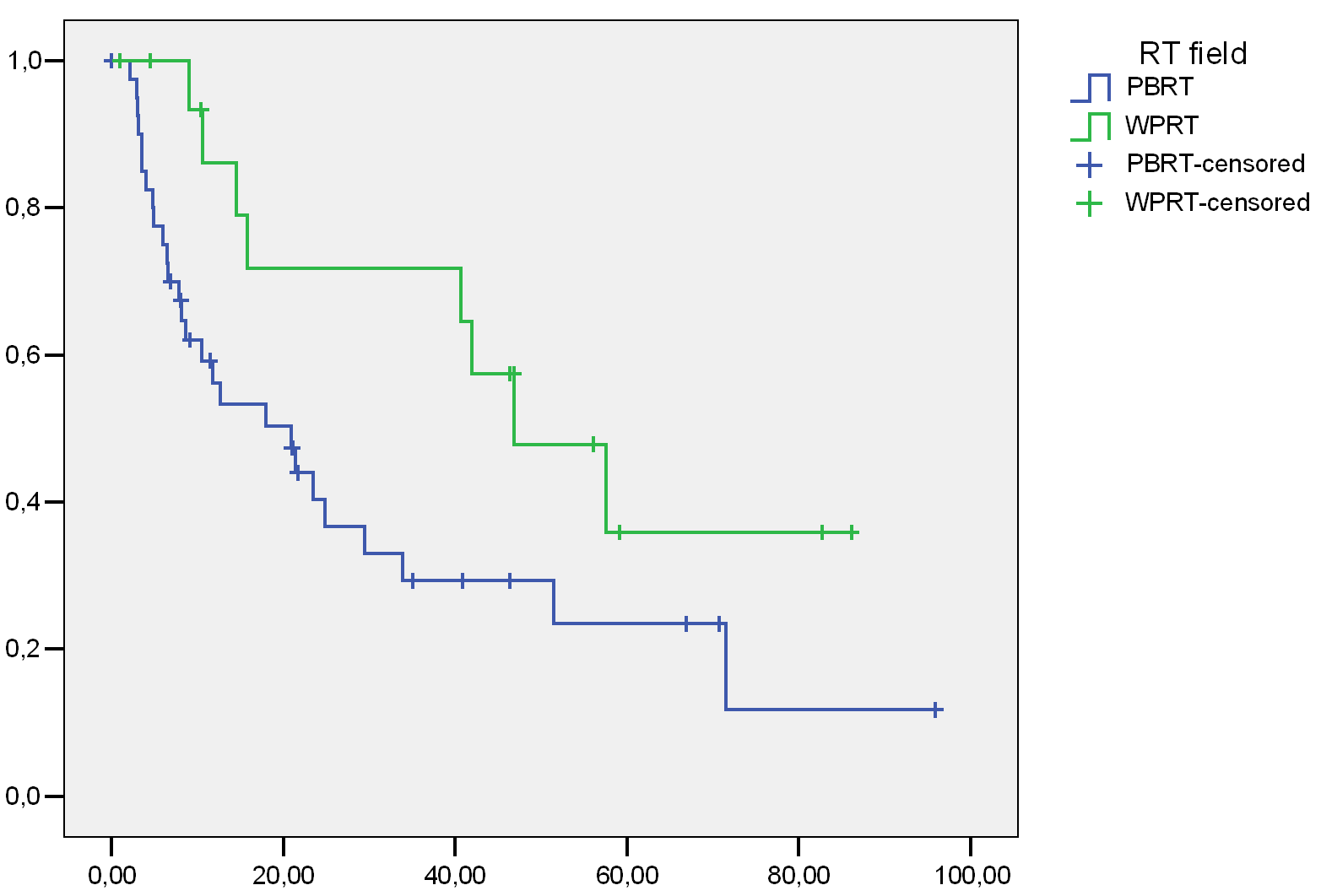


**Freedom from biochemical failure (FFBF) in patients who had salvage whole pelvis radiotherapy (WPRT) compared to fossa-only radiotherapy (PBRT)**

**Months**

**FFBF**

| **No. at risk** |  |  |  |  |  |  |  |
| --- | --- | --- | --- | --- | --- | --- | --- |
| **Months** | **0** | **20** | **40** | **60** | **80** | **100** | **120** |
| **Whole pelvis (WPRT)** | 17 | 10 | 10 | 2 | 2 | 0 | 0 |
| **Fossa-only (PBRT)** | 41 | 17 | 7 | 4 | 1 | 0 | 0 |

Pelvic_RT: 0=PBRT; 1=WPRT

c)


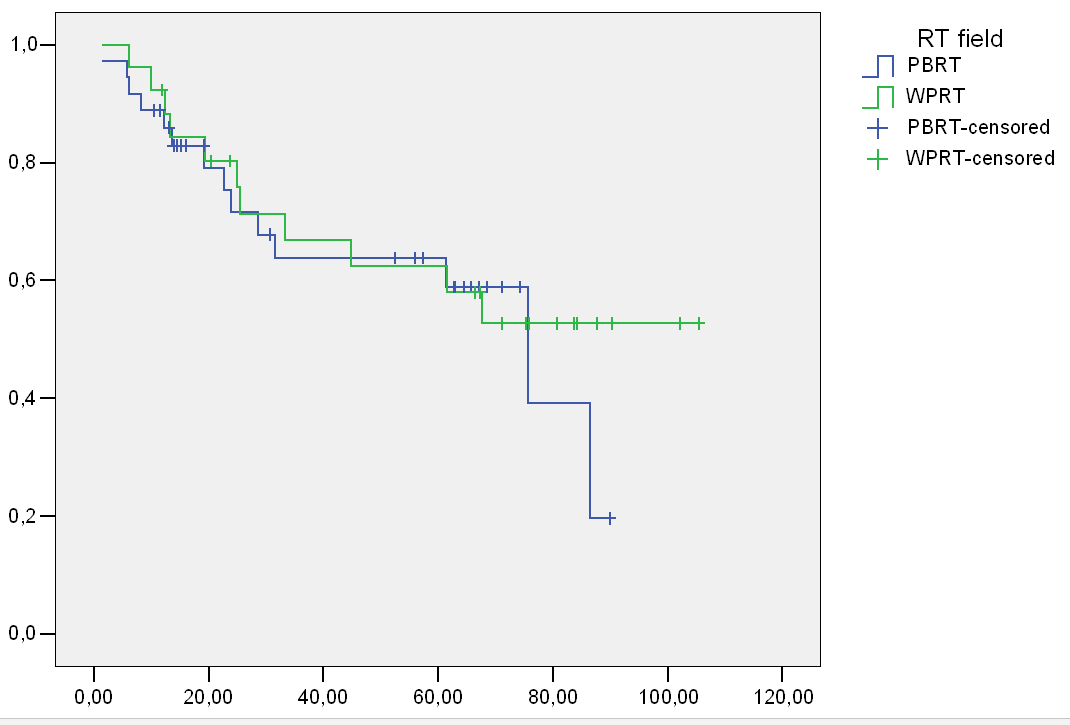


**Biochemical progression-free survival (bPFS) in patients who had postoperative (but not salvage) radiotherapy with whole pelvis radiotherapy (WPRT) compared to fossa-only irradiation (PBRT)**

**Months**

**bPFS**

| **No. at risk** |  |  |  |  |  |  |  |
| --- | --- | --- | --- | --- | --- | --- | --- |
| **Months** | **0** | **20** | **40** | **60** | **80** | **100** | **120** |
| **Whole pelvis (WPRT)** | 26 | 20 | 15 | 14 | 7 | 2 | 0 |
| **Fossa-only (PBRT)** | 36 | 21 | 16 | 13 | 2 | 0 | 0 |

Pelvic_RT: 0=PBRT; 1=WPRT

d)


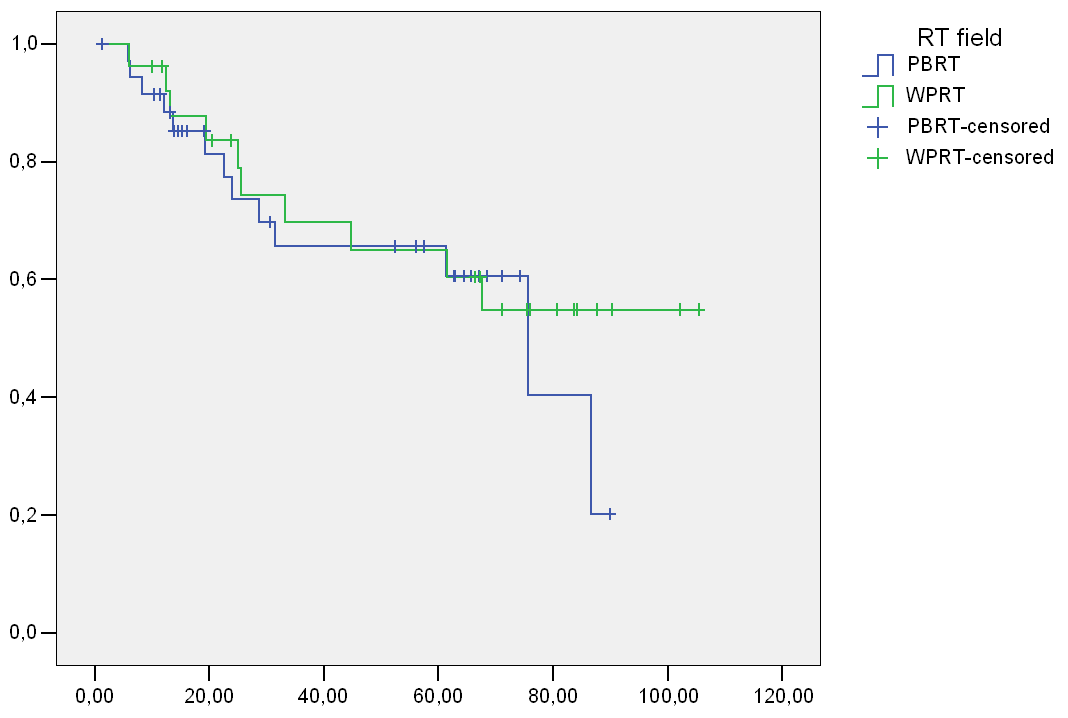


**Freedom from biochemical failure (FFBF) in patients who had postoperative (but not salvage) radiotherapy with whole pelvis radiotherapy (WPRT) compared to fossa-only irradiation (PBRT)**

**Months**

**FFBF**

| **No. at risk** |  |  |  |  |  |  |  |
| --- | --- | --- | --- | --- | --- | --- | --- |
| **Months** | **0** | **20** | **40** | **60** | **80** | **100** | **120** |
| **Whole pelvis (WPRT)** | 26 | 20 | 15 | 14 | 7 | 2 | 0 |
| **Fossa-only (PBRT)** | 36 | 21 | 16 | 13 | 2 | 0 | 0 |

Pelvic_RT: 0=PBRT; 1=WPRT
