# Supplementary material for: Postoperative elective pelvic nodal irradiation compared to prostate bed irradiation in locally advanced prostate cancer – a retrospective analysis of dose-escalated patients
Source: Radiat Oncol. 2019 Jun 7;14:96. doi: 10.1186/s13014-019-1301-5 (PMC6554899; doi:10.1186/s13014-019-1301-5)
Supplement: Supplementary file 6 — Figure S6. Univariate survival analyses comparing FFBF and bPFS between the analysed cohort of patients with locally advanced tumors and patients with localized tumors and node-positive tumors at our department. (DOCX 373 kb) [file 13014_2019_1301_MOESM6_ESM.docx]

**Supplementary figure S-6**

a)

**Biochemical progression-free survival (bPFS) in patients, who had locally advanced tumors (main dataset; n=120) compared to patients who had localized tumors (n=52); irrespective of WPRT or PBRT**

**Months**

**bPFS**


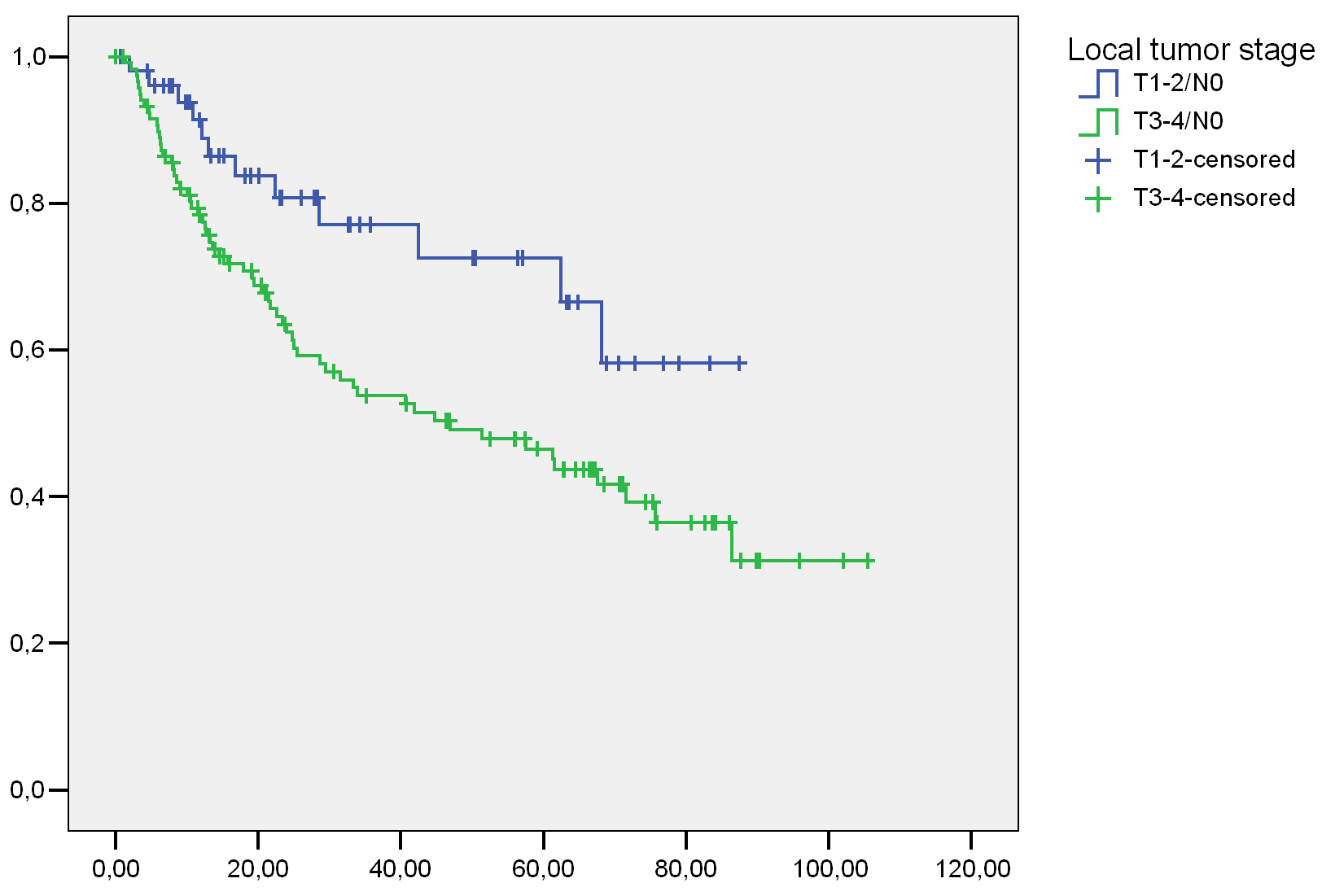


| **No. at risk** |  |  |  |  |  |  |  |
| --- | --- | --- | --- | --- | --- | --- | --- |
| **Months** | **0** | **20** | **40** | **60** | **80** | **100** | **120** |
| **Localized tumors** | 52 | 29 | 17 | 12 | 2 | 0 | 0 |
| **Locally advanced tumors** | 120 | 68 | 48 | 33 | 12 | 2 | 0 |

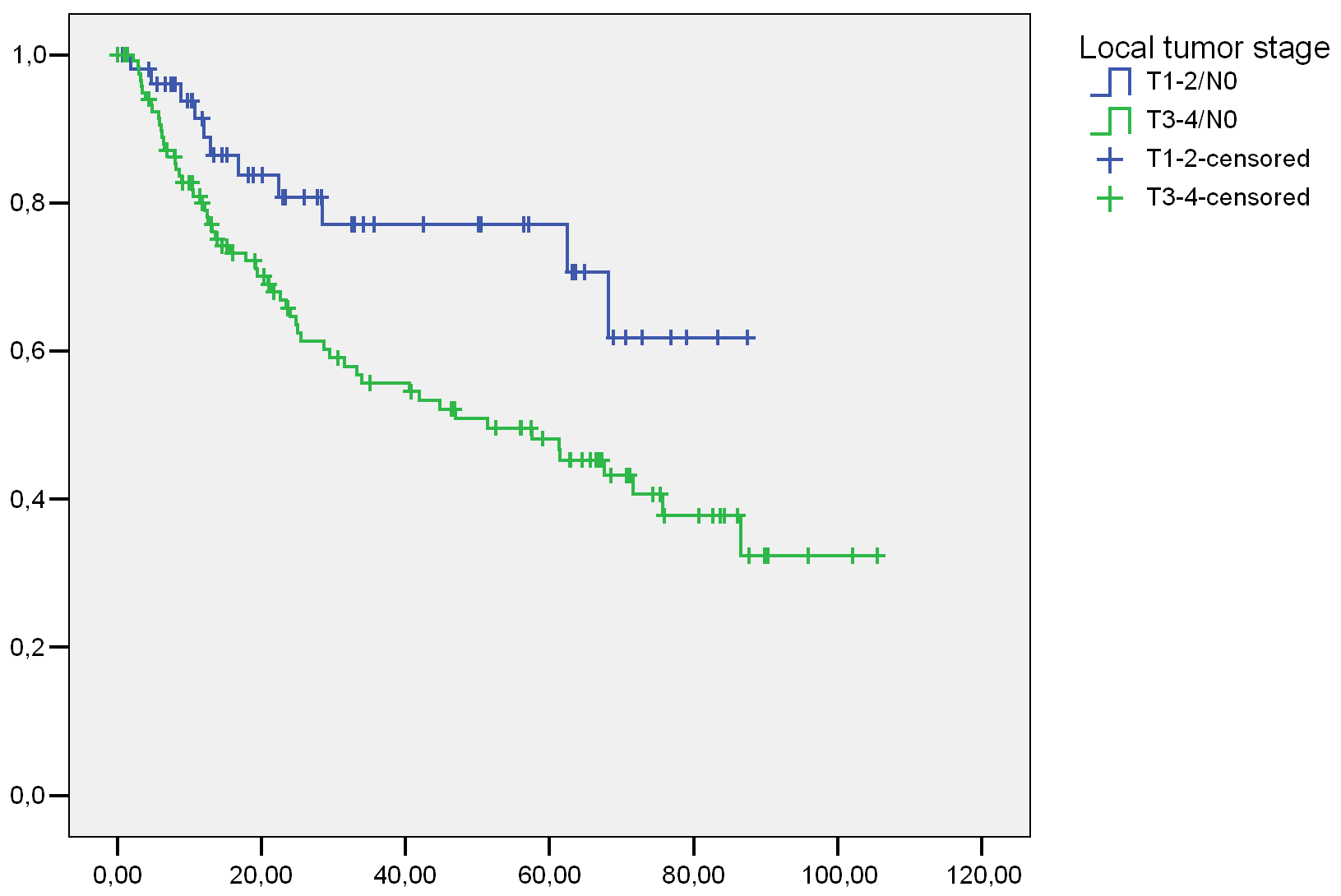
b)

**Biochemical progression-free survival (FFBF) in patients, who had locally advanced tumors (main dataset; n=120) compared to patients who had localized tumors (n=52); irrespective of WPRT or PBRT**

**Months**

**FFBF**

| **No. at risk** |  |  |  |  |  |  |  |  |
| --- | --- | --- | --- | --- | --- | --- | --- | --- |
| **Months** | | **0** | **20** | **40** | **60** | **80** | **100** | **120** |
| **Localized tumors** | | 52 | 29 | 17 | 12 | 2 | 0 | 0 |
| **Locally advanced tumors** | | 120 | 68 | 48 | 33 | 12 | 2 | 0 |

c)


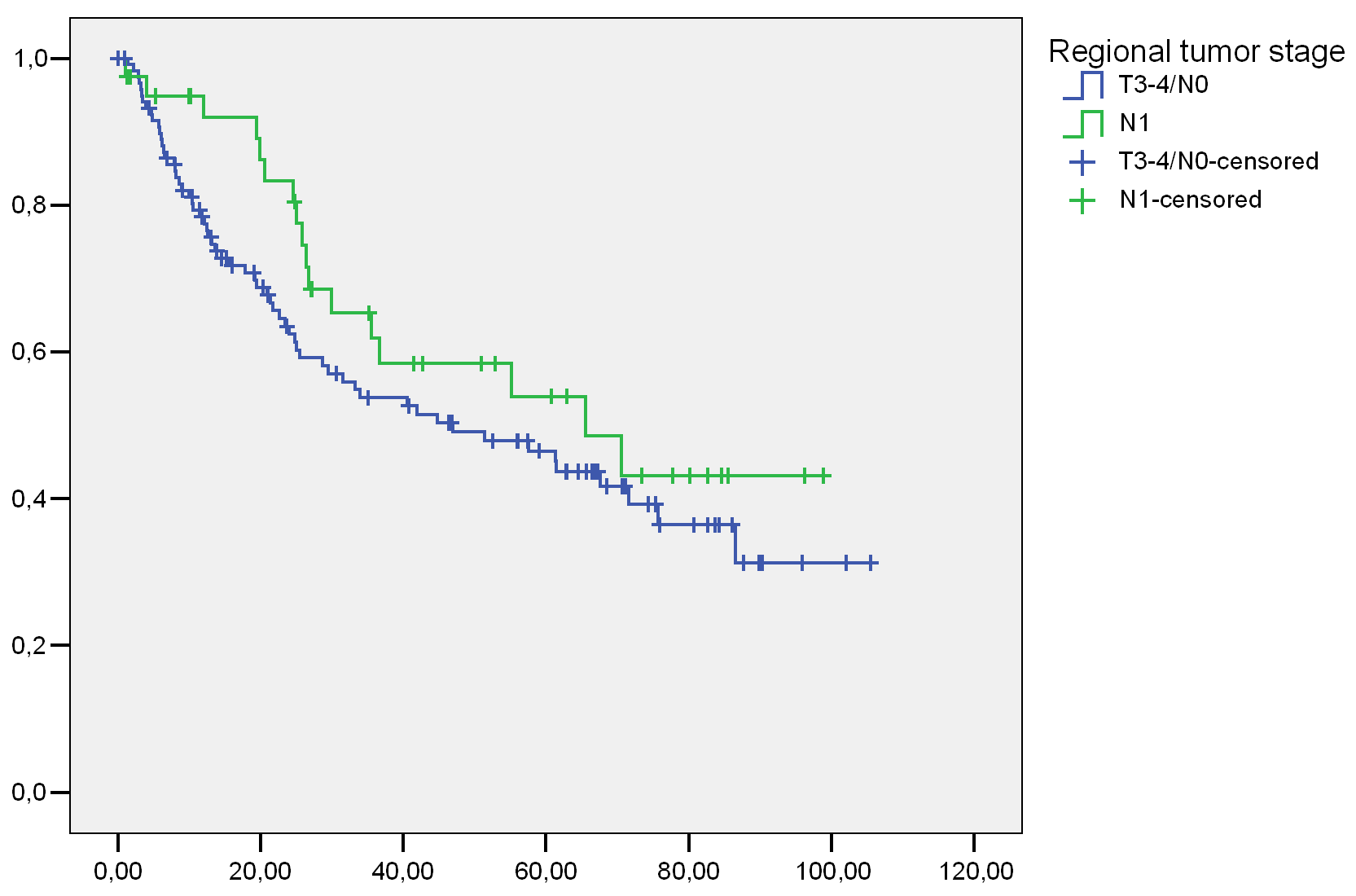


**Biochemical progression-free survival (bPFS) in patients, who had locally advanced tumors (main dataset; n=120; T3-4/N0) compared to patients who had node-positive tumors (n=40; T1‑4/N1)**

**Months**

**bPFS**

| **No. at risk** |  |  |  |  |  |  |  |
| --- | --- | --- | --- | --- | --- | --- | --- |
| **Months** | **0** | **20** | **40** | **60** | **80** | **100** | **120** |
| **Locally advanced tumors** | 120 | 68 | 48 | 33 | 12 | 2 | 0 |
| **Node-positive tumors** | 40 | 30 | 17 | 12 | 6 | 0 | 0 |

d)**
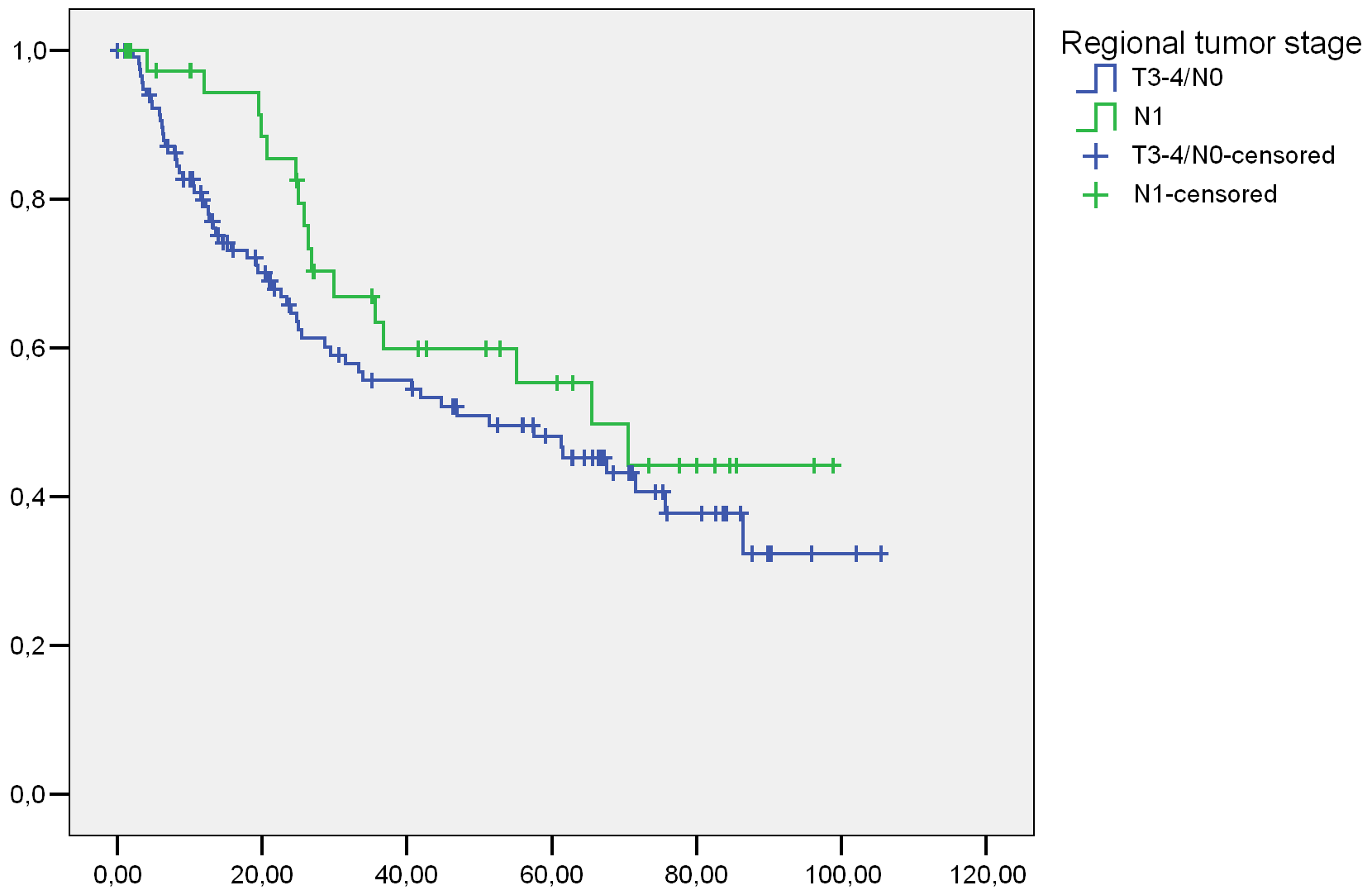
**

**Biochemical progression-free survival (FFBF) in patients, who had locally advanced tumors (main dataset; n=120; T3-4/N0) compared to patients who had node-positive tumors (n=40; T1‑4/N1)**

**Months**

**FFBF**

| **No. at risk** |  |  |  |  |  |  |  |
| --- | --- | --- | --- | --- | --- | --- | --- |
| **Months** | **0** | **20** | **40** | **60** | **80** | **100** | **120** |
| **Locally advanced tumors** | 120 | 68 | 48 | 33 | 12 | 2 | 0 |
| **Node-positive tumors** | 40 | 30 | 17 | 12 | 6 | 0 | 0 |

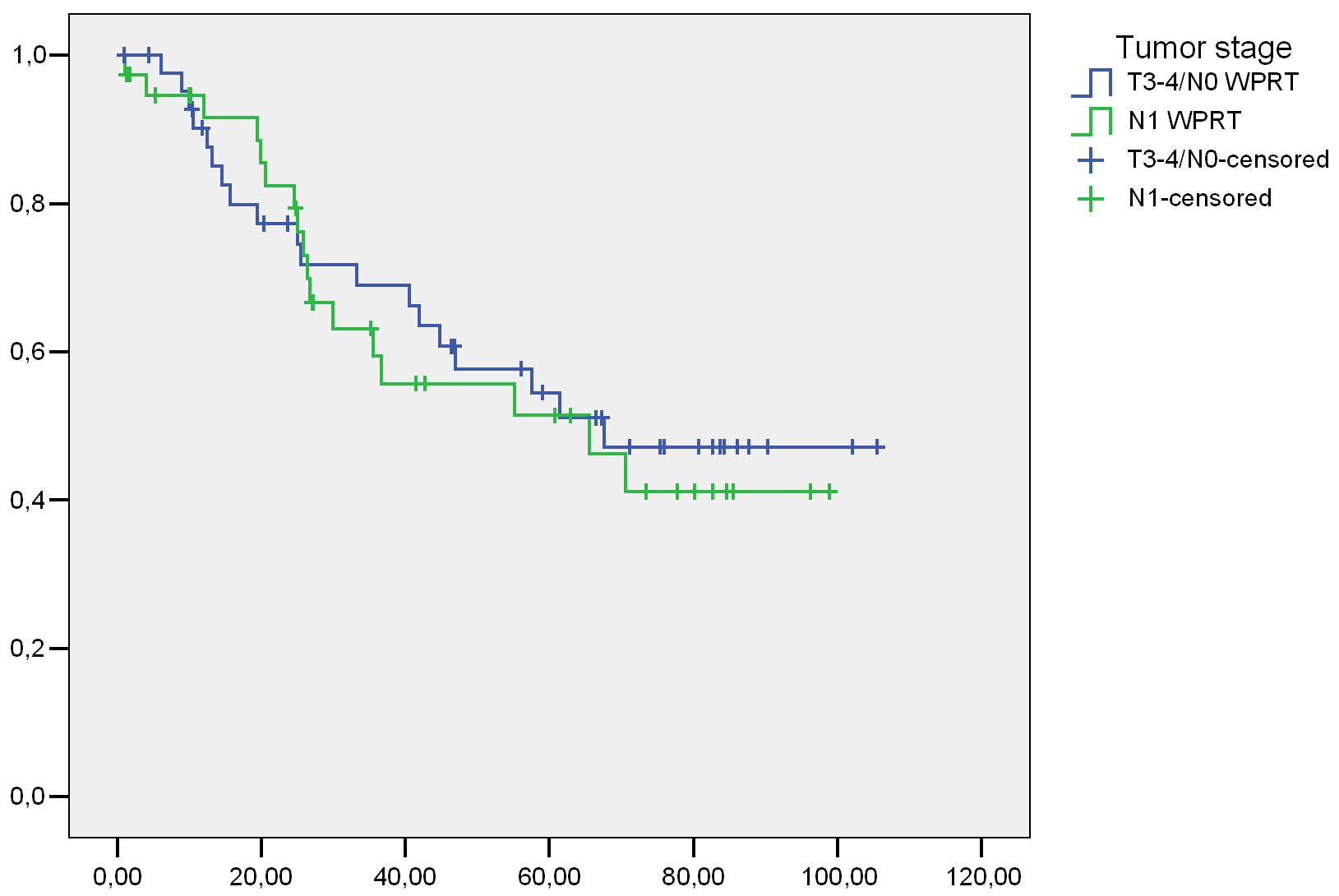
e)

**Patients with T3-4/N0 tumor showed numerically (albeit not significantly) worse outcomes compared to patients with node-positive tumors (see previous curves SF-6c-d); we assumed that the imbalance of WPRT usage (N1: 95% vs. T3-4/N0: 35.8%) might have contributed; therefore, we additionally compared T3-4/N0-WPRT with T1-4/N1-WPRT**

**Months**

**bPFS**

| **No. at risk** |  |  |  |  |  |  |  |
| --- | --- | --- | --- | --- | --- | --- | --- |
| **Months** | **0** | **20** | **40** | **60** | **80** | **100** | **120** |
| **T3-4/N0 treated w. WPRT** | 43 | 30 | 25 | 16 | 9 | 2 | 0 |
| **T1-4/N0 treated w. WPRT** | 38 | 28 | 15 | 12 | 6 | 0 | 0 |

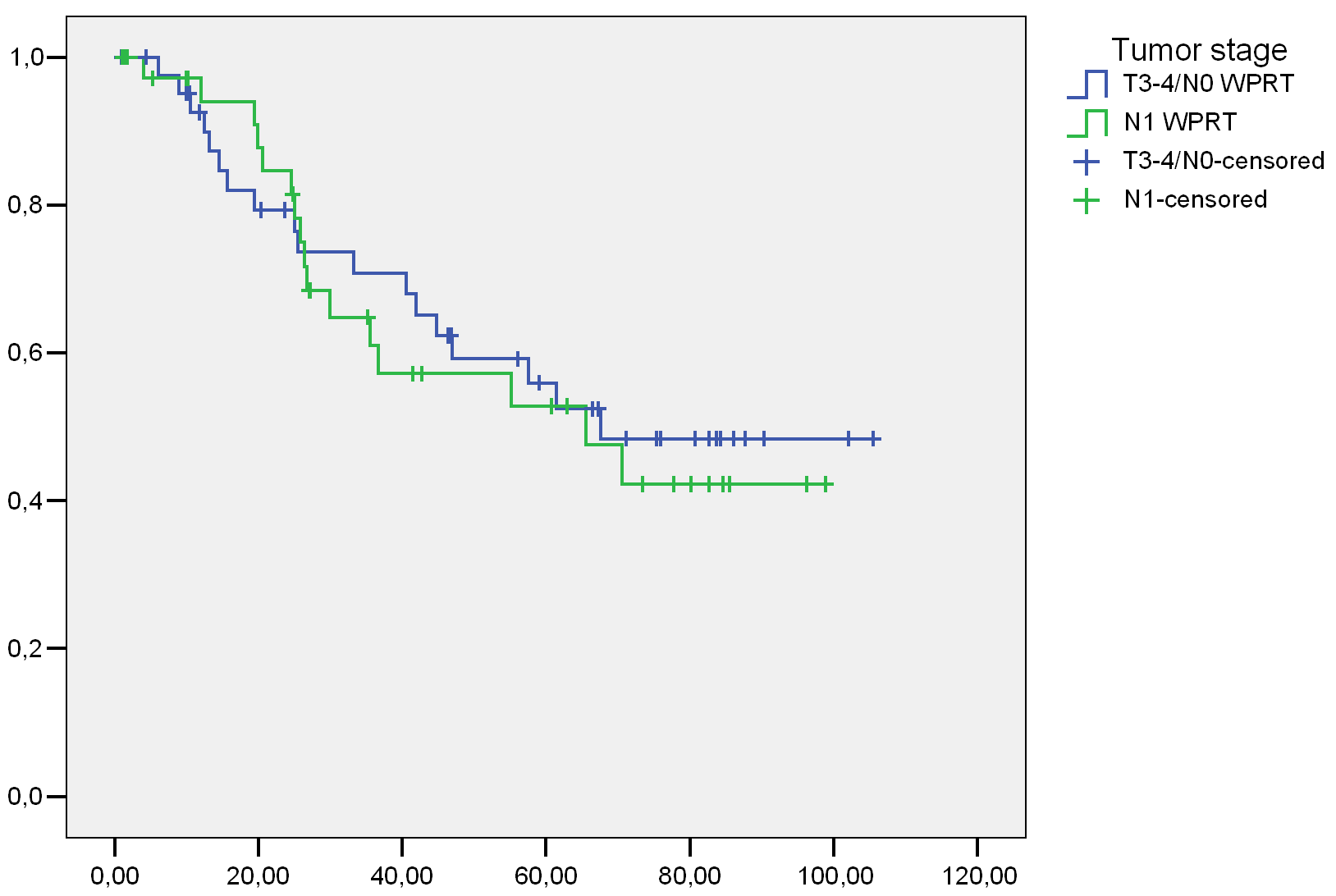
f)

**Patients with T3-4/N0 tumor showed numerically (albeit not significantly) worse outcomes compared to patients with node-positive tumors (see previous curves SF-6c-d); we assumed that the imbalance of WPRT usage (N1: 95% vs. T3-4/N0: 35.8%) might have contributed; therefore, we additionally compared T3-4/N0-WPRT with T1-4/N1-WPRT**

**Months**

**FFBF**

| **No. at risk** |  |  |  |  |  |  |  |
| --- | --- | --- | --- | --- | --- | --- | --- |
| **Months** | **0** | **20** | **40** | **60** | **80** | **100** | **120** |
| **T3-4/N0 treated w. WPRT** | 43 | 30 | 25 | 16 | 9 | 2 | 0 |
| **T1-4/N0 treated w. WPRT** | 38 | 28 | 15 | 12 | 6 | 0 | 0 |
