# Supplementary material for: Postoperative elective pelvic nodal irradiation compared to prostate bed irradiation in locally advanced prostate cancer – a retrospective analysis of dose-escalated patients
Source: Radiat Oncol. 2019 Jun 7;14:96. doi: 10.1186/s13014-019-1301-5 (PMC6554899; doi:10.1186/s13014-019-1301-5)
Supplement: Supplementary file 7 — Table S1. This file shows multivariate models which include patients with localized disease and/or patients with node-positive tumors. (DOC 76 kb) [file 13014_2019_1301_MOESM7_ESM.doc]

**Supplementary table S-1a:** Multivariate survival model for biochemical progression-free survival (bPFS); this analysis includes all patients with localized disease or locally advanced disease treated after prostatectomy with the specified treatment protocol at our department.

| **Variable** | **Hazard Ratio** | **95% CI** | ***P*** |
| --- | --- | --- | --- |
| Elective pelvic radiotherapy (WPRT) | 0.550 | 0.319-0.949 | 0.032 |
| Detectable tumor and high-dose radiotherapy | 0.504 | 0.319-0.949 | 0.033 |
| Salvage radiotherapy | 1.134 | 0.579-2.218 | 0.714 |
| Androgen-deprivation therapy (ADT-) usage | 0.691 | 0.284-1.681 | 0.415 |
| PSA values at start of radiotherapy above 0.2 ng/ml | 1.649 | 0.984-2.764 | 0.058 |
| Higher Gleason score of surgical specimens | 1.538 | 1.192-2.025 | 0.001 |
| Locally advanced ^*^ | 2.152 | 1.084-4.272 | 0.029 |

* Locally advanced was introduced to account for the univariate association of shorter bPFS and FFBF (both p<0.05) with locally advanced compared to localized tumors.

**Supplementary table S-1b:** Multivariate survival model for freedom from biochemical failure (FFBF); this analysis includes all patients with localized disease or locally advanced disease treated after prostatectomy with the specified treatment at our department.

| **Variable** | **Hazard Ratio** | **95% CI** | ***P*** |
| --- | --- | --- | --- |
| Elective pelvic radiotherapy (WPRT) | 0.570 | 0.326-0.997 | 0.049 |
| Detectable tumor and high-dose radiotherapy | 0.470 | 0.245-0.900 | 0.023 |
| Salvage radiotherapy | 1.121 | 0.561-2.242 | 0.746 |
| Androgen-deprivation therapy (ADT-) usage | 0.600 | 0.229-1.573 | 0.299 |
| PSA values at start of radiotherapy above 0.2 ng/ml | 1.745 | 1.021-2.981 | 0.042 |
| Higher Gleason score of surgical specimens | 1.505 | 1.146-1.976 | 0.003 |
| Locally advanced ^*^ | 2.305 | 1.132-4.695 | 0.021 |

* Locally advanced was introduced to account for the univariate association of shorter bPFS and FFBF (both p<0.05) with locally advanced compared to localized tumors.

**Supplementary table S-1c:** Multivariate survival model for biochemical progression-free survival (bPFS); this analysis includes all patients treated at our department with the described radiation therapy approach who did not have distant metastases at time of irradiation (T1-4/N0-1; M0; n=212).

| **Variable** | **Hazard Ratio** | **95% CI** | ***P*** |
| --- | --- | --- | --- |
| Elective pelvic radiotherapy (WPRT) | 0.558 | 0.348-0.895 | 0.015 |
| Detectable tumor and high-dose radiotherapy | 0.526 | 0.302-0.916 | 0.023 |
| Salvage radiotherapy | 0.974 | 0.546 -1.738 | 0.929 |
| Androgen-deprivation therapy (ADT-) usage | 0.602 | 0.279-1.301 | 0.197 |
| PSA values at start of radiotherapy above 0.2 ng/ml | 1.710 | 1.069-2.736 | 0.025 |
| Higher Gleason score of surgical specimens | 1.555 | 1.213-1.993 | 0.001 |
| Localized or loco/regionally advanced ^*^ | 1.977 | 1.003-3.898 | 0.049 |

* Patients with node-positive tumors did not have significantly or numerically worse outcomes in univariate models compared to locally advanced but node-negative tumors; in contrast, univariate analysis showed numerically better outcomes for node-positive (T1-4/N1), compared to locally advanced tumor patients (T3-4/N0); possibly due to a higher usage of WPRT in node-positive patients (95%) compared to patients with node-negative locally advanced disease (35.8%). When only patients treated with WPRT were compared, there was a slight numerical improvement for T3-4/N0 compared to T1-4/N1 patients (not significant), suggesting that WPRT may have compensated for the higher risk associated with positive lymph nodes. Details are shown in supplementary figures SF-6c-f. Because of the similar outcomes between T3-4/N0 and N1 tumor patients, we grouped T1-4/N1 patients in the same category as T3-4/N0 patients and only distinguished between localized vs. (all) advanced tumors. Introducing a separate category for regionally advanced tumors (T1-4/N1) did not change the main result that WPRT, high-dose radiotherapy/detectable disease, higher pre-radiotherapy PSA values and Gleason scores were independently associated with bPFS.

**Supplementary table S-1d:** Multivariate survival model for freedom from biochemical failure (FFBF); this analysis includes all patients treated at our department with the described radiation therapy approach who did not have distant metastases at time of irradiation (T1‑4/N0-1; M0; n=212).

| **Variable** | **Hazard Ratio** | **95% CI** | ***P*** |
| --- | --- | --- | --- |
| Elective pelvic radiotherapy (WPRT) | 0.574 | 0.354-0.932 | 0.025 |
| Detectable tumor and high-dose radiotherapy | 0.516 | 0.292-0.912 | 0.023 |
| Salvage radiotherapy | 0.958 | 0.528 -1.738 | 0.888 |
| Androgen-deprivation therapy (ADT-) usage | 0.466 | 0.195-1.115 | 0.086 |
| PSA values at start of radiotherapy above 0.2 ng/ml | 1.776 | 1.093-2.884 | 0.020 |
| Higher Gleason score of surgical specimens | 1.524 | 1.181-1.966 | 0.001 |
| Localized or loco/regionally advanced ^*^ | 2.107 | 1.042-4.261 | 0.038 |

* Patients with node-positive tumors did not have significantly or numerically worse outcomes in univariate models compared to locally advanced but node-negative tumors; in contrast, univariate analysis showed numerically better outcomes for node-positive (T1-4/N1), compared to locally advanced tumor patients (T3-4/N0); possibly due to a higher usage of WPRT in node-positive patients (95%) compared to patients with node-negative locally advanced disease (35.8%). When only patients treated with WPRT were compared, there was a slight numerical improvement for T3-4/N0 compared to T1-4/N1 patients (not significant), suggesting that WPRT may have compensated for the higher risk associated with positive lymph nodes. Details are shown in supplementary figures SF-6c-f. Because of the similar outcomes between T3-4/N0 and N1 tumor patients, we grouped T1-4/N1 patients in the same category as T3-4/N0 patients and only distinguished between localized vs. (all) advanced tumors. Introducing a separate category for regionally advanced tumors (T1-4/N1) did not change the main result that WPRT, high-dose radiotherapy/detectable disease, higher pre-radiotherapy PSA values and Gleason scores were independently associated with FFBF.
